# GliaMorph: a modular image analysis toolkit to quantify Müller glial cell morphology

**DOI:** 10.1242/dev.201008

**Published:** 2023-02-02

**Authors:** Elisabeth Kugler, Isabel Bravo, Xhuljana Durmishi, Stefania Marcotti, Sara Beqiri, Alicia Carrington, Brian Stramer, Pierre Mattar, Ryan B. MacDonald

**Affiliations:** ^1^ Institute of Ophthalmology, University College London, 11-43 Bath St, Greater London EC1V 9EL, UK; ^2^ Randall Centre for Cell & Molecular Biophysics, King's College London, New Hunt's House, London SE1 1UL, UK; ^3^ Department of Cellular and Molecular Medicine, University of Ottawa, Ottawa, ON, K1H 8M5, Canada; ^4^ Ottawa Hospital Research Institute (OHRI), Ottawa, ON, K1H 8L6, Canada

**Keywords:** Retina, Müller glia, Glia morphology, Zebrafish, Mouse, Development

## Abstract

Cell morphology is crucial for all cell functions. This is particularly true for glial cells as they rely on complex shape to contact and support neurons. However, methods to quantify complex glial cell shape accurately and reproducibly are lacking. To address this, we developed the image analysis pipeline ‘GliaMorph’. GliaMorph is a modular analysis toolkit developed to perform (1) image pre-processing, (2) semi-automatic region-of-interest selection, (3) apicobasal texture analysis, (4) glia segmentation, and (5) cell feature quantification. Müller glia (MG) have a stereotypic shape linked to their maturation and physiological status. Here, we characterized MG on three levels: (1) global image-level, (2) apicobasal texture, and (3) regional apicobasal vertical-to-horizontal alignment. Using GliaMorph, we quantified MG development on a global and single-cell level, showing increased feature elaboration and subcellular morphological rearrangement in the zebrafish retina. As proof of principle, we analysed expression changes in a mouse glaucoma model, identifying subcellular protein localization changes in MG. Together, these data demonstrate that GliaMorph enables an in-depth understanding of MG morphology in the developing and diseased retina.

## Teaser

**Summary:** GliaMorph allows for multi-dimensional Muller glial cell analysis, enabling image-level as well as subcellular morphological assessments that are robust, adaptable and easy to use.

## INTRODUCTION

Although a vast amount of biomedical research relies on microscopy data and image-driven research, methods to process cell morphology objectively and reproducibly are lacking. However, computational analysis is paramount to understanding cell function and connectivity on a more abstract level, particularly in complex tissues. Glial cells are some of the most morphologically elaborate cells (MacDonald et al., 2017; Wang et al., 2017) and provide a myriad of functions in the central nervous system (CNS) (Oberheim et al., 2009; Khakh and Sofroniew, 2015). To fulfil these crucial functions, glial cells are precisely shaped to contact neurons, synapses and the vasculature. Glial shape is not only pivotal in healthy tissue, but is altered in numerous neurodegenerative conditions, and can precede neuronal dysfunction in some cases such as epilepsy (Sharma et al., 2019) or diabetic retinopathy (Lasta et al., 2013). Hence, measuring glial morphology in a robust and reliable manner is paramount to our understanding of CNS development and dysfunction.

Current methods for glial cell morphological analysis often require user input (i.e. manual cell tracing) that might result in subjective bias, offer crude measurements (i.e. not subcellular resolution and not reproducible detail), or are challenging to adapt to specific biological questions and dynamic time-lapse acquisitions. This leads to a data analysis bottleneck in morphological analysis and image-based cell profiling (Pennisi, 2016; Fetz et al., 2016). As such, it is necessary to develop workflows and high-quality datasets of glial morphologies for robust and (semi-)automatic analysis of glial shape in healthy and diseased CNS. In the era of machine learning, defining relevant features could be assumed to be easily achievable. However, machine-learning methods require appropriate training sets or *a priori* information. Therefore, conventional image analysis workflows are required to establish benchmark datasets before machine-learning approaches could be considered. Hence, there is a rationale to develop computational, (semi-)automatic analysis methods to resolve glial cell morphology and status (Escartin et al., 2021).

As a part of the CNS, the retina serves as a tractable model to study cell morphology and structure due to its highly stereotypic architecture. The retina consists of seven main cell types: six neuronal and one glial cell type called Müller glia (MG). MG are radial glia that are considered molecular and functional homologues to astrocytes (Masland, 2012), as they carry out numerous physiological roles to support neurons (Newman and Reichenbach, 1996; Bringmann et al., 2006) and emanate elaborate fine projections to contact synapses (Wang et al., 2017). Nascent MG cells derive from retinal progenitor cells, beginning as simple radial cells that then mature to morphologically elaborate cells with a highly branched morphology (MacDonald et al., 2017; Williams et al., 2010). The mature MG are organized laterally such that they interact with each other in a so-called ‘tiled’ fashion, contacting almost all cells in the retina (MacDonald et al., 2017; Wang et al., 2017). Thus, MG morphology is linked to their spatial and functional organization. Moreover, it was shown that MG shape is indicative of their maturity (MacDonald et al., 2017) and health (Halford et al., 2017). For instance, neuronal tissue damage can elicit MG to undergo gliosis, a reactive state whereby their morphology and gene expression levels are drastically altered; this is observed in several diseases, such as glaucoma (Seitz et al., 2013).

**Fig. 1.**
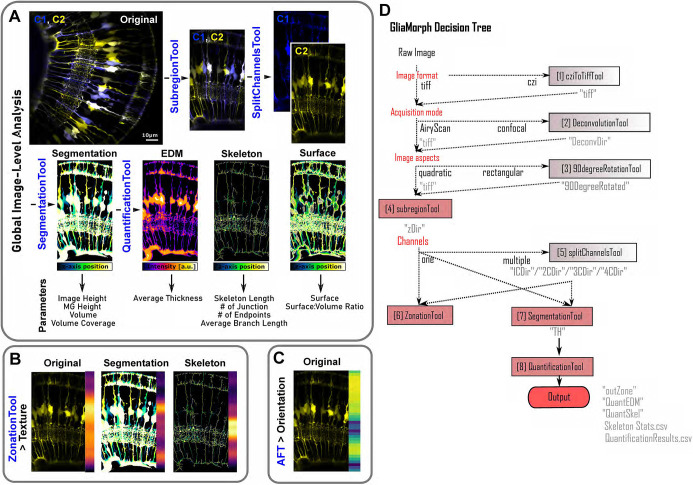
**GliaMorph workflow overview.** (A) Using global image-level measurements, we developed a tool to quantify 11 different parameters of MG morphology. (B) Using the zonationTool and applying it to original, segmented and skeletonized data allows insights into apicobasal subcellular feature distributions. (C) Fourier transformation-based analysis allows the assessment of apicobasal subcellular orientation distributions. (D) As GliaMorph is modular in its application, workflow design is easy and flexible to suit user needs. AFT, alignment by Fourier transform; EDM, Euclidean distance map.

Here, we establish a workflow, called ‘GliaMorph’, that allows for the reproducible assessment of glia shape (Fig. 1). GliaMorph is a 3D image analysis toolkit to describe quantitatively the stereotypic cellular morphology of MG. Specifically, we present (1) an in-depth description of encountered data challenges, (2) steps for image pre-processing to improve data quality; (3) a novel tool for semi-automatic region-of-interest (ROI) selection to allow comparability between samples and groups, (4) a method to plot apicobasal textures automatically, (5) an MG segmentation workflow, and (6) a workflow for 3D quantification of glia. This allows for image assessment at three levels: global image level, apicobasal texture, and apicobasal vertical-to-horizontal alignment. We apply this to retinas of fully transgenic zebrafish, in which all MG cells are labelled, and to mosaic-injected embryos, in which individual MG cells are labelled. We show that MG become significantly more morphologically complex throughout their maturation from 60 h post fertilization (hpf) to 96 hpf in the zebrafish retina. Finally, we apply GliaMorph to immunohistochemistry data from the mouse glaucoma model DBA/2J (Turner et al., 2017) and detect signs of gliosis, demonstrating that this tool has the potential to work across species and identify subcellular pathological changes in MG. Taken together, our work provides a benchmark for 3D MG analysis across MG visualization techniques, developmental ages, and species.

## RESULTS

### Data understanding informs the design of a computational workflow to assay MG cell morphology

**Fig. 2.**
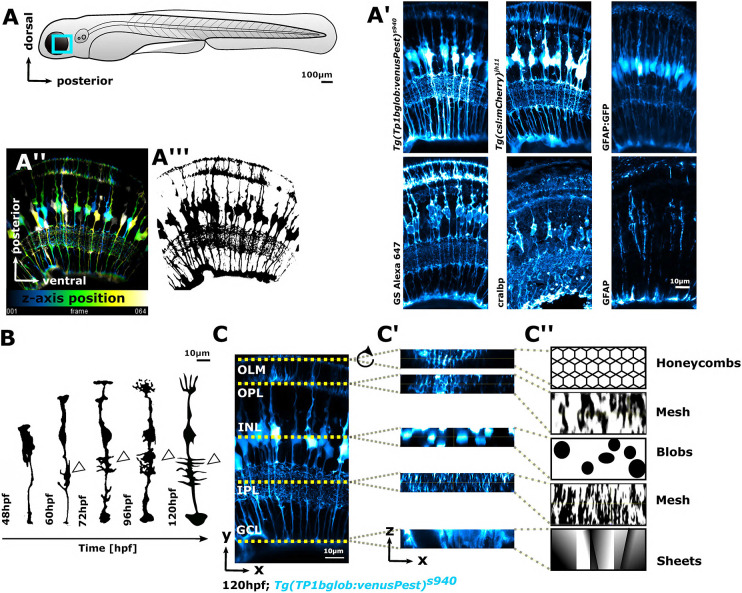
**MG cells have a complex shape that makes them challenging to analyse computationally.** (A) Imaging of MG was conducted in the ventro-temporal zebrafish retina to standardize the ROI. (A′) MG can be visualized with a variety of transgenic reporter lines or immunohistochemistry markers. (A″) Depth-coded MIP of MG stable transgenic line. (A‴) Data as in A″ after image segmentation. (B) Hand-drawn schematic of individual MG cell morphological maturation during early development, showing elaboration of subregions and an increase in protrusions (arrowheads) based on observed biological data (MacDonald et al., 2017). (C) MIP micrograph with yellow dotted lines indicating the apicobasal position of retinal layers. (C′) Resliced/transformed sections at the position of the yellow dotted lines, illustrating that cell subregions have cellularly and computationally distinct properties along the apicobasal axis. (C″) Manually drawn schematic of how MG subregions features could be described for computational analysis in terms of shape. In all images, apical is the top and basal is the bottom of the image. INL, inner nuclear layer; OLM, outer limiting membrane.

Zebrafish are a well-established model for studying retina development and disease (Richardson et al., 2017; Gestri et al., 2012; Angueyra and Kindt, 2018; Malicki et al., 2016). The overall organization and composition of the retina, including MG function and structure, are highly conserved between zebrafish and human (Richardson et al., 2017; O'Brown et al., 2018). Furthermore, the zebrafish retina is suitable for morphological analysis as it is accessible for advanced imaging, rapidly develops, and is amenable to various manipulation and/or visualization techniques. These include specific transgenic reporter lines (Vázquez-Chona et al., 2009) and antibodies labelling MG markers (e.g. Glutamine synthetase, GS) (Fig. 2A-A‴). However, as these labels mark a considerable proportion of MG in the retina, it is challenging, both morphologically and computationally, to identify or quantify individual cells (Fig. 2A″). Furthermore, MG become increasingly morphologically elaborate during retinal development (Fig. 2B). Therefore, the zebrafish retina is suitable for generating the high-resolution imaging data required to develop a computational workflow to quantify complex glial morphologies robustly in the healthy and diseased vertebrate retina.

When developing image quantification approaches for cell morphology, it is essential to understand data to being able to establish computational analysis workflows. This is particularly important when working with complex data, such as images of MG cells, as their shape is complex and changes along the apicobasal axis (Fig. 2A-A‴). To allow for sufficient resolution to resolve individual glial subdomains, we used confocal imaging (Jonkman et al., 2020). First, we focused on the optimization of data acquisition and data quality as this greatly impacts downstream analysis outcomes. To standardize the ROI for image acquisition, we focused on the ventro-temporal retina, as regional differences in anatomy (e.g. high-acuity area versus periphery) and cell morphologies (e.g. photoreceptor neurons) exist across the retina in zebrafish (Fig. 2A-A‴) (Zhou et al., 2020; Yoshimatsu et al., 2020). MG have five apico-basal subregions and each fulfils a specific function for nearby retinal neurons (MacDonald et al., 2017) (Fig. 2C). Computationally these regions fall into categories based on their geometries. This is seen more clearly when looking at cross-sections of the 3D data stack (Fig. 2C′). It also becomes clear that MG have complex morphologies and that different subregions pose different computational challenges (Fig. 2C″). Subregion 1 resembles a honeycomb structure, allowing MG to interact with photoreceptors. Subregion 2 resembles a fine mesh-like structure, allowing MG to interweave with synaptic terminal of photoreceptors, bipolar and horizontal cells. Subregion 3 contains blob-like cell bodies. Subregion 4 is characterized by mesh-like protrusions in the inner plexiform layer (IPL), and subregion 5 contains the sheet-like endfeet. In addition to this apical-to-basal differences, these subregions are also computationally distinct with respect to signal levels and patterns with our transgenic and antibody markers (Fig. S1A). As such, we considered imaging parameters such as 3D signal intensity profiles for MG markers (Fig. S1B,C), optimized sampling frequency (Fig. S1E) and examined potential imaging artefacts (e.g. blurring; Fig. S1F-H). This data understanding allowed us to determine the required image processing steps and optimize data acquisition, resulting in the GliaMorph data analysis workflow presented here (Fig. 1). To ensure accessibility of the method, all steps were implemented in the open-source image analysis software Fiji (Schindelin et al., 2012).

### Establishing image comparability and reproducibility to quantify MG cells reliably in the retina

**Fig. 3.**
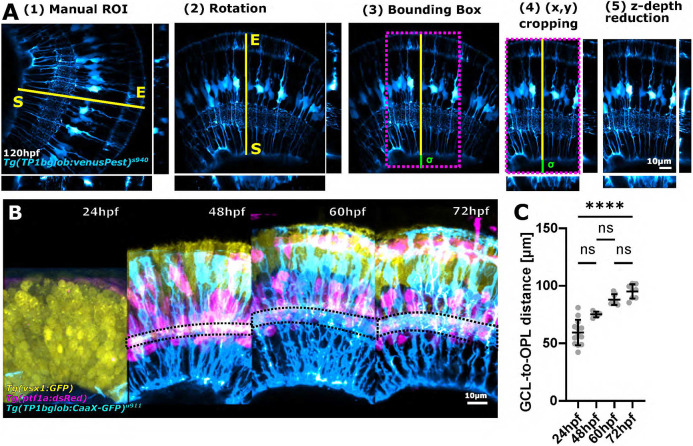
**Image standardization facilitates global MG measurements across developmental time.** (A) Workflow of the subregionTool. Semi-automatic subregion selection by (1) manual line ROI (yellow line) selection, which is then used to rotate the image (2), create a bounding box (3), crop the image using this bounding box (magenta dotted box) (4), and crop the image in the *z* dimension (5) (sigma=user-defined basal extension to allow for blood vessel inclusion). (B) Images from different animals and different transgenic reporter lines (representative images) overlaid with each other after processing with the subregionTool. (C) Measurement of GCL-to-OPL distance in age-matched samples [measured in *Tg(TP1bglob:CaaX-GFP)^u911^*; CoV 24 hpf 18.72%, 48 hpf 3.13%, 60 hpf 5.25%, 72 hpf 6.52%; *P*<0.0001; 24 hpf *n*=13 embryos, 48 hpf *n*=5 embryos, 60 hpf *n*=8 embryos, 72 hpf *n*=8 embryos; *N*=2 experimental repeats; Kruskal–Wallis test; mean±s.d.]. ns, not significant.

For robust characterization of glial morphology, imaging datasets must first be processed identically to allow images to be compared within and between groups. For example, images may differ in sample orientation, acquisition position, *z*-axis size differences, or field of view (FOV). Thus, images cannot be directly compared to each other but require pre-processing to allow comparable ROIs between samples. To establish image similarity, we developed the semi-automatic ‘subregionTool’, which enables ROI extraction based on manual line selection (Fig. 3A), and can be applied to right or left eyes (Fig. S2A,B). Once the ROI is selected, the subregionTool automatically (1) aligns them along the *y*-axis (Fig. S2C,D), (2) creates a bounding box to crop images in the *xy* dimension (see Fig. S2E,F and code for details), and then (3) reduces the stack to the specified depth (Fig. S2G). As the subregionTool is also applicable to other imaging datasets, not only MG, we were able to overlap data from different neuronal markers, as well as images from 24-72 hpf including neurons as well as MG (Fig. 3B). To confirm data comparability, we measured progenitor/MG height, revealing high similarity in age-matched samples, and that the developmental growth of MG is highly consistent [Fig. 3C; coefficient of variation (CoV) 24 hpf 18.72%, 48 hpf 3.13%, 60 hpf 5.25%, 72 hpf 6.52%; *P*<0.0001]. Together, these data show that the subregionTool allows the establishment of reproducible 3D images, enabling images to be compared objectively, making sample and group assessments possible.

When performing fluorescence microscopy, images do not directly reproduce the object of interest owing to artefacts as well as the system impulse function or convolution of light, called point spread function (PSF) (de Gennaro, 2010). To restore object properties before data processing and object measurements, a deconvolution step is typically performed. We established the ‘deconvolutionTool’, which allows processing of data acquired in confocal mode (Fig. S3). To establish an easy-to-use deconvolution approach, we integrated existing plugins into the deconvolutionTool to allow single-/multi-channel input, selection of fluorophore wavelengths, different objective numerical aperture (NA), and theoretical or experimental PSF file input (Fig. 1). We found that 3D deconvolution outperforms 2D deconvolution when imaging MG in transgenic zebrafish, resulting in reduced background (Fig. S4, white arrowheads), and high deconvolution results in increased structured noise and grains (Fig. S4, black arrowheads). Together, this emphasizes that the imaging modality (e.g. confocal versus Airyscan) and parameters of deconvolution influence data quality for subsequent data analysis.

### Global MG apicobasal structure can be visualized by 1D vector analysis

**Fig. 4.**
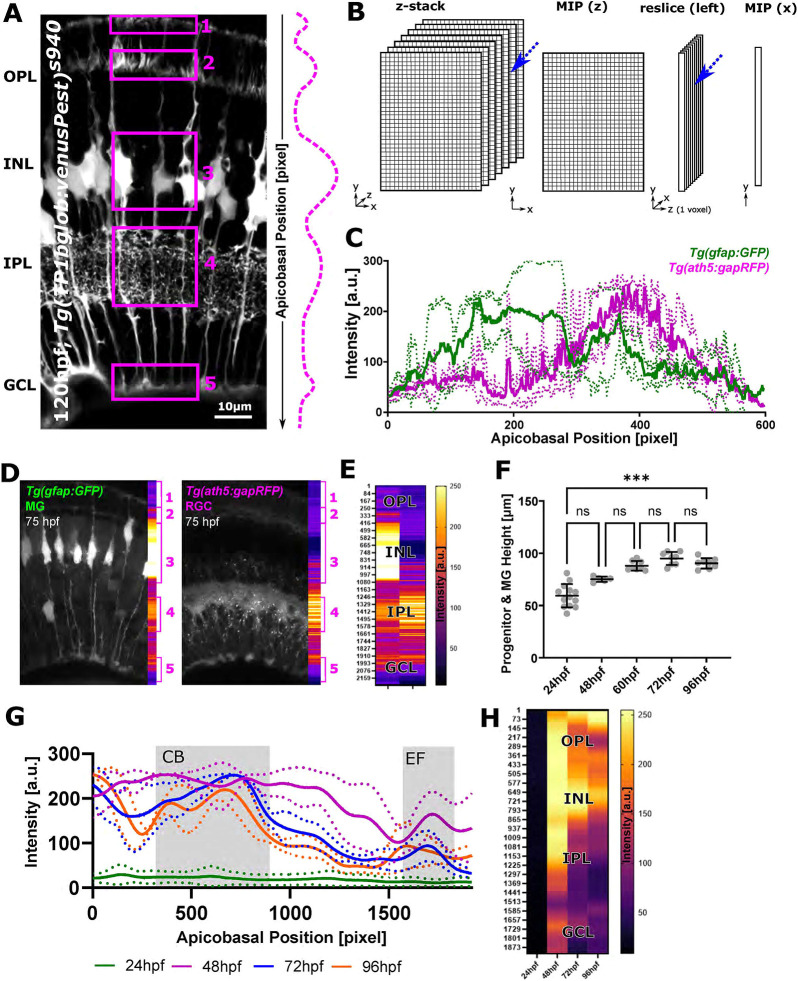
**The zonationTool enables the identification of distinct apicobasal MG subregions.** (A) MG morphological specializations from the apical (top) to basal (bottom) position in the retina. Subregions 1-5 are highlighted by boxes. Dashed line indicates assumption of relative intensity profile of cells from apical to basal. (B) Diagram of the workflow: 3D image stacks were reduced to 2D images, transformed to 1D+*z*, and again reduced, thus resulting in a one-voxel-wise representation of MG data. (C) Apicobasal intensity plot derived using the zonationTool (as described in B) of the double-transgenic *Tg(GFAP:GFP); Tg(ath5:RFP)* at 75 hpf. Apicobasal position is absolute in pixels (normalization presented in the following). (D) Representative images showing differences between transgenic lines. (E) Heatmap representation for apicobasal texture analysis of the images shown in D (MG, left; RGC, right). (F) Retina height measurements (or GCL-to-OPL distance) from 24 to 96 hpf shows a statistically significant increase over time [***P=0.0006; not significant (ns) *P*>0.999; 24 hpf *n*=13 embryos, 48 hpf *n*=5 embryos, 60 hpf *n*=8 embryos, 72 hpf *n*=8 embryos, 96 hpf *n*=8 embryos; *N*=2 experimental repeats; Kruskal–Wallis test; mean±s.d.]. (G) Intensity profiles from 24 to 96 hpf produced with the zonationTool (solid line depicting mean values; image size normalized to 1900 pixels for comparability). (H) Heatmap representation for apicobasal texture analysis of the data shown in G (mean). a.u., arbitrary unit; CB, cell bodies; EF, endfeet; INL, inner nuclear layer; RGC, retinal ganglion cells.

As MG have a stereotypic apicobasal pattern with differential distribution of geometries and intensity (Fig. 4A), we next wanted to utilize this to describe 3D MG data (*x*,*y*,*z*) in a simplified form (1D vector) using dimensionality reduction. To reduce data, we developed the ‘zonationTool’ (Fig. 4B, Table 1), which reduces data first in the *z*-axis (2D; *x*,*y*), transforms them by 90° (2D; *y*,*z*), and then performs another dimensionality reduction (1D; *y*). This dimensionality reduction allows assessment of the intensity distributions or ‘zonation’ in the apical-to-basal direction across the retina. As the approach is again independent of input data, it can be applied to different labelling approaches of MG as well as other cell types, such as retinal neurons (Fig. 4C-F). We next wanted to examine MG zonation or texture from 24 to 96 hpf, but first needed to understand these in more depth, i.e. whether MG at different developmental stages are comparable in size and how this changes over time. Measuring the distance between the granule cell and outer plexiform layers (GCL-to-OPL distance) (also progenitor or MG height), a significant increase was found over time (*P*=0.0006, Kruskal–Wallis test; Fig. 4F). When analysing the CoV, variation was low, suggesting comparability between samples, meaning we could align 1D vectors of age-matched samples without processing. Briefly, the highest CoV was observed at 24 hpf 18.72%, with lower CoV values at 48 hpf 3.31%, 60 hpf 5.25%, 72 hpf 6.52% and 96 hpf 5.46%. In addition to this, we wanted to assess how local texture changed from 24 to 96 hpf. To do this, we normalized the apicobasal axis to 1920 pixels to allow direct visual comparison. This showed that endfeet are identifiable from 48 hpf onwards, and a clear discrimination between MG cell bodies and the IPL is possible from 72 hpf onwards (Fig. 4G,H). Together, dimensionality reduction of 3D images using the zonationTool is a user-friendly way to visualize texture and subregional zones of MG as 1D vectors.

**Table 1.**
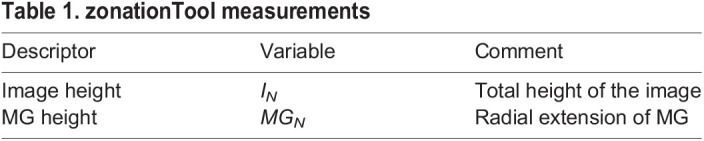
zonationTool measurements

### 3D feature extraction reveals MG subcellular elaboration during development

**Fig. 5.**
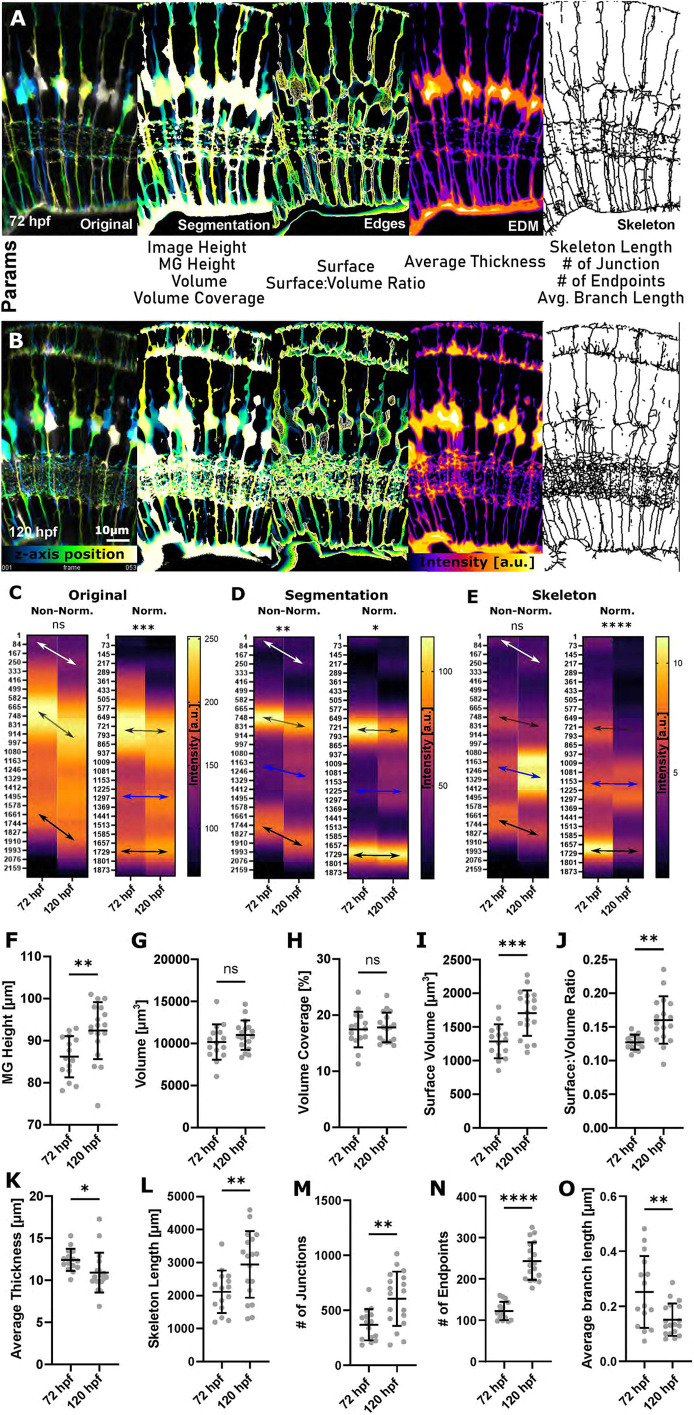
**3D quantification of MG using GliaMorph shows that MG significantly elaborate between 72 hpf and 120 hpf.** (A,B) Workflow overview to extract MG features from a cytosolic transgenic on a 3D global image level, including depth-coded (DC) original images, DC segmentation, DC surface, thickness (EDM; higher intensity represents thicker regions) and skeleton (MIP dilated for representation) at 72 hpf (A) and 120 hpf (B). a.u., arbitrary unit. (C) Apicobasal texture plot of original images showing changes in subregions 1 and 2 (white arrow), cell bodies (grey arrow) and endfeet (*P*=0.0862). Normalization refers to image length, i.e. both images were adjusted to the same length. (D) Apicobasal texture plot of segmented images indicates IPL maturation (blue arrows) (**P=0.0052). (E) Apicobasal texture plot of skeletonized images over time (*P*=0.3402; Mann–Whitney *U*-test; mean). *P*-values for C-E refer to non-normalized data. (F) MG height was significantly increased from 72 to 120 hpf (**P=0.0061). (G) Volume was not significantly changed (*P*=0.2314). (H) Volume coverage was not significantly changed (*P*>0.9999). (I) Surface volume was significantly increased (***P=0.0004). (J) Surface-to-volume ratio was statistically significantly increased (**P=0.0015). (K) Average thickness was significantly decreased (*P=0.0366). (L) Skeleton length was statistically significantly increased (**P=0.0100). (M) The number of junctions was significantly increased (**P=0.0026). (N) The number of endpoints was significantly increased (****P<0.0001). (O) Average branch length was significantly changed (**P=0.0060). 72 hpf *n*=15, 120 hpf *n*=18; *N*=2 experimental repeats; two-tailed, unpaired *t*-test; mean±s.d. ns, not significant.

We used *Tg(TP1bglob:VenusPest)^s94^*, an established transgenic reporter line for visualizing MG, to develop and test the ability of the GliaMorph toolkit to quantify 3D cell features. To examine whether MG features became more elaborate with maturity, we analysed data at 72 hpf and 120 hpf. After using the subregionTool for data comparability, to extract MG in the images, we established the ‘segmentationTool’, which uses bleach correction, 8-bit conversion, 3D median filtering, and Otsu-based thresholding to produce binary/segmented images. We then applied the ‘quantificationTool’, which extracts the following global image-level features from the segmented image: (1) image height: length of *y*-axis, (2) MG volume: voxels classified as MG after segmentation, and (3) density: ratio of total image voxels divided by MG volume voxels (i.e. given as a fraction of 1). After surface extraction using Canny edge detection, (4) surface area is quantified. Using 3D thinning, the skeleton/centreline was extracted to quantify (5) network length, (6) number of branching points, (7) number of end points, and (8) average branch length. Lastly, combining the skeleton with a 3D Euclidean Distance Map, (9) the average thickness was analysed (Fig. 5A,B, Table 2). Applying the zonationTool to plot the apicobasal texture of MGs showed growth of MG and downward migration of nuclei (Fig. 5C; cell subdomain 3). To also assess for local patterns, we normalized data, which confirmed an elaboration of subdomains with maturation (Fig. 5C-E). Applying the zonationTool to the segmented data showed again an increase in size and MG cell bodies that were positioned more basally at 120 hpf. Also, clear bands of apical MG zones (1 and 2) and endfeet were seen (Fig. 5D; subdomain 5). When plotting the automatically skeletonized images, elaborations of MG from 72 to 120 hpf were pronounced in the IPL (Fig. 5E; subdomain 4).

**Table 2.**
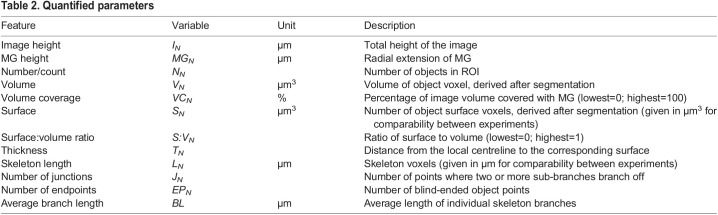
Quantified parameters

Following this apicobasal analysis, we quantified specific MG 3D features with the quantificationTool at 72 hpf and 120 hpf. Quantification of MG height showed a significant increase from 72 to 120 hpf (*P*=0.0061; Fig. 5F). Surprisingly, neither MG volume (*P*=0.2314; Fig. 5G) nor percentage volume coverage were significantly different between 72 and 120 hpf (*P*>0.9999; Fig. 5H). However, MG surface volume (*P*=0.0004; Fig. 5I) and surface-to-volume ratio were significantly increased from 72 to 120 hpf (*P*=0.0015; Fig. 5J), suggesting that shape complexity increased over time. The average thickness was significantly decreased from 72 to 120 hpf (*P*=0.0366; Fig. 5K), which was thought to be due to an increase in the number of thinner protrusions over time. As expected, skeleton length (*P*=0.0100; Fig. 5L), number of junctions (*P*=0.0026; Fig. 5M) and number of endpoints (*P*<0.0001; Fig. 5N) were significantly increased and average branch length was decreased from 72 to 120 hpf (*P*=0.0060; Fig. 5O). Together, these data show that GliaMorph is suitable for assessing MG morphology in complete transgenic retinas along the apicobasal axis as well as in 3D to extract biologically meaningful information.

### Visualization of membrane-tagged fluorophores supersedes cytosolic reporters in quantification of MG morphology

As cellular labels are used to visualize the glial cells and their gross morphology, we must consider their differing characteristics for robust analysis (e.g. cytosolic versus membrane expression) (Escartin et al., 2021; Halford et al., 2017). As GliaMorph analysis is based on object intensity and distribution, we next compared MG-specific transgenic lines expressing the cytosolic fluorescent protein *Tg(csl:mCherry)^jh11^* with mosaic single-cell expression of the membrane fluorescent marker transgene *Tg(TP1glob:CaaX-GFP)^u911^* (72 hpf; Figs S5, S6A-C). Visually, the membrane marker delineated more detail than the cytosol marker, as seen for MG protrusions in the IPL (Fig. S6A-C, white arrowheads) or MG honeycombing (Nagashima et al., 2017) in the outer limiting membrane (Fig. S6A-C, unfilled arrowhead). A tested segmentation approach delivered satisfying outcomes with the membrane marker, but not the cytosol marker (Fig. S6D-F). Moreover, with the membrane marker cell connectivity and IPL protrusion details were extracted. This was also reflected in the 3D skeleton, which showed more detail with the membrane marker (Fig. S6G-I). Together, we conclude that membrane visualization leads to more accurate visualization of morphology.

### MG development is defined by apicobasal elaboration and refinement of subcellular domains

**Fig. 6.**
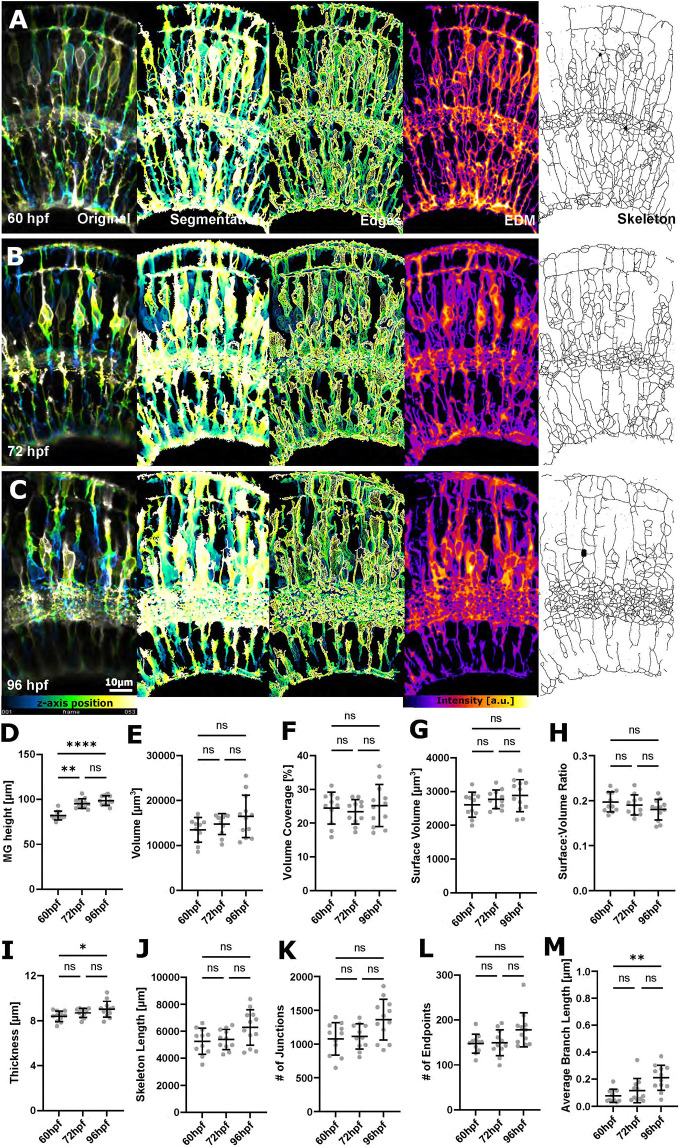
**Increased MG feature extraction using membrane-tagged fluorescent reporter lines.** (A-C) Micrographs of original and processed data at 60, 72 and 96 hpf. a.u., arbitrary unit. (D) MG height was statistically significant increased from 60 to 96 hpf (****P<0.0001). (E) Volume was not statistically significantly changed (*P*=0.2197). (F) Volume coverage was not statistically significantly changed (*P*=0.7728). (G) Surface volume was not statistically significantly changed (*P*=0.3036). (H) Surface-to-volume ratio was not statistically significantly changed (*P*=0.3570). (I) Thickness was statistically significantly increased from 60 to 96 hpf (*P=0.0466). (J) Skeleton length was not statistically significantly changed (*P*=0.1095). (K) The number of junctions was not statistically significantly changed (*P*=0.0741). (L) The number of endpoints was not statistically significantly changed (*P*=0.0690). (M) Average branch length was statistically significantly increased from 60 to 96 hpf (**P=0.0018). 60 hpf *n*=11, 72 hpf *n*=12, 96 hpf *n*=13; *N*=2 experimental repeats; Kruskal–Wallis test; mean±s.d. All *P*-values refer to the comparison of the 60 and 96 hpf time points.

**Fig. 7.**
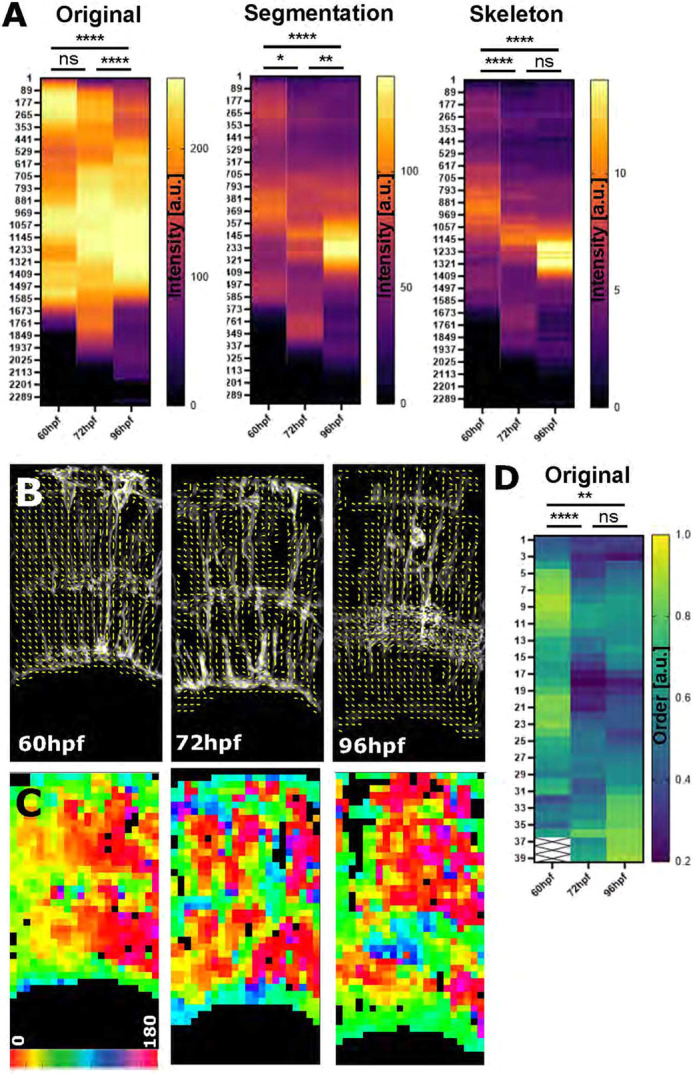
**Zonation and orientation measurements identify increased subcellular organization of MG cell processes during development.** (A) Apicobasal intensity plotting using the zonationTool showed a statistically significant difference from 60 to 96 hpf in original (****P<0.0001), segmented (****P<0.0001) and skeletonized images (****P<0.0001) (60 hpf *n*=11, 72 hpf *n*=12, 96 hpf *n*=13; *N*=2 experimental repeats; Kruskal–Wallis test; mean). (B) Orientation measurement using Fourier transformation analysis delivered local orientation (yellow lines) in non-zero (black) regions. (C) Image as in B but colour-coded for orientation, showing that subcellular organization changed from a more vertical (1, yellow) to a more horizontal (0.2, blue) alignment. (D) Image order collapsed into 1D vectors for quantitative comparison (*P*=0.0049 for the comparison of the 60 and 96 hpf time points; data from two experimental repeats; Kruskal–Wallis test). a.u., arbitrary unit; ns, not significant.

As membrane labels outperformed cytosolic MG cell labelling from single-cell labelling experiments, we generated the stable transgenic line *Tg(TP1bglob:eGFP-CAAX)^u911^.* Using this, we analysed MG development in a shorter time frame from 60 to 96 hpf (Fig. 6A-C). This revealed statistically significant increases in MG height (*P*<0.0001; Fig. 6D), thickness (*P*=0.0466; Fig. 6I), and average branch length (*P*=0.0018; Fig. 6M), but none of the other measured parameters (Fig. 6E-H,J-L). This led us to examine our data in a local fashion using apicobasal distributions. This revealed a significant difference in intensities from 60 to 96 hpf for original (*P*<0.0001), segmented (*P*<0.0001) and skeletonized images (*P*<0.0001; Fig. 7A). We observed a downward migration of nuclei, increased MG height, and increased overall complexity (as indicated by skeleton distributions), particularly in the IPL. We then examined the alignment of structures in the image (i.e. horizontal versus vertical), which can be described as image order (Marcotti et al., 2021) (Fig. 7B,C), revealing a significant difference from 60 to 96 hpf (*P*=0.0049; Fig. 7D). These data suggest that even though features might not change enough to be extracted globally (Fig. 6), local features are elaborated and refined over time. When comparing our 72 hpf data of the cytosolic transgenic *Tg(TP1bglob:venusPest)* with the membrane-tagged transgenic *Tg(TP1bglob:eGFP-CAAX)^u911^*, most quantified features were increased in the membrane-tagged transgenic.

### Single-cell analysis validates the global image-level measurements of MG morphology

**Fig. 8.**
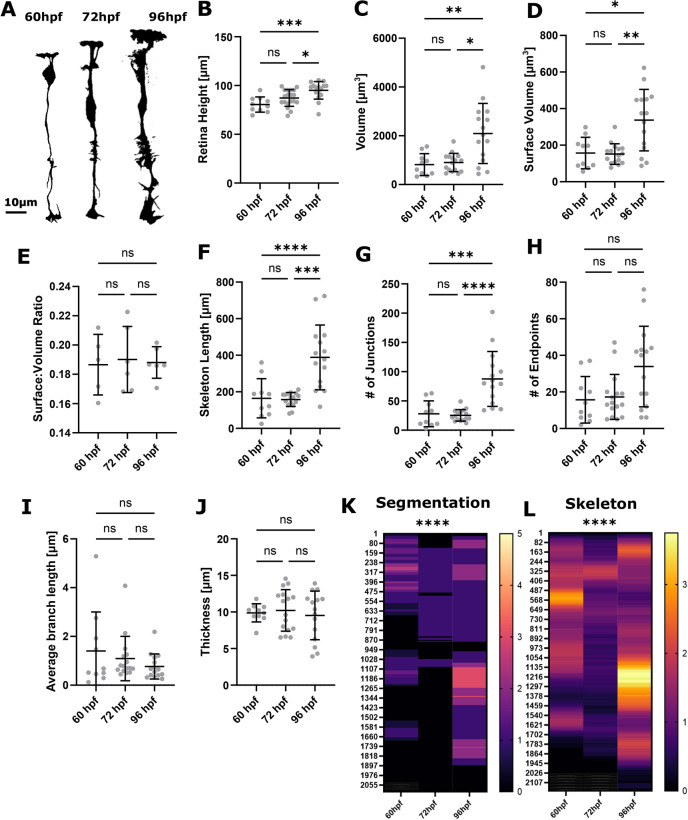
**Analysis of MG development using single-cell measurements reveals variability between cells.** (A) Segmentation MIPs of single MG cells at 60 hpf, 72 hpf and 96 hpf (representative images extracted from 3D stacks). (B) MG height did significantly increase from 60 to 96 hpf (***P=0.0006). (C) Volume did significantly increase from 60 to 96 hpf (**P=0.0035). (D) Surface volume did significantly increase from 60 to 96 hpf (*P=0.0029). (E) Surface-to-volume ratio did not significantly change from 60 to 96 hpf (*P*=0.9947). (F) Skeleton length did significantly increase from 60 to 96 hpf (**P<0.0001). (G) The number of junctions did significantly increase from 60 to 96 hpf (***P<0.0001). (H) The number of endpoints did not significantly alter from 60 to 96 hpf (*P*=0.0400). (I) Average branch length did not significantly alter from 60 to 96 hpf (*P*=0.3320). (J) Thickness did not significantly alter from 60 to 96 hpf (*P*=0.8241). 60 hpf *n*=10 cells, 72 hpf *n*=19 cells, 96 hpf *n*=16 cells; *N*=3 experimental repeats; Kruskal–Wallis test; mean±s.d. (K,L) Apicobasal intensity plotting using the zonationTool showed a statistically significant difference from 60 to 96 hpf in segmented (****P<0.0001) and skeletonized (****P<0.0001) images.

As the above measurements are based on the population-level (image-level or global) analysis, we next sought to study cell heterogeneity and whether measurements of individual cells represent collective measurements of cell populations. Thus, we visualized and analysed data from individual MG cells from 60 to 96 hpf (Fig. 8A). In individual MGs, we saw changes in retina height, surface-to-volume ratio, skeleton length, and endpoints (Fig. 8B,E,F,H) just as we observed at the population level (Fig. 5D,H,I,L). Other parameters, such as volume, surface, and number of junctions were increased in single-cell level measurements (Fig. 8C,D,G), whereas branch length and thickness were not altered at the single-cell level (Fig. 8I,J). Together, our data show that single-cell and global image-level measurements are in good agreement, also with respect to zonationTool-based analysis (Fig. 8K,L), but that batch effects might impact measurement outcomes. For example, single-cell thickness measurements showed high variability, which was not observed for global image-level measurements. Conversely, using single-cell measurements allows for a closer examination of cellular heterogeneity. Although precise measurements can be derived from single-cell analysis, the sampling problem they introduce becomes important. This highlights that global and single-cell analysis might answer different biological questions.

### Apicobasal feature analysis provides reliable readouts of MG defects in a mouse model of glaucoma

**Fig. 9.**
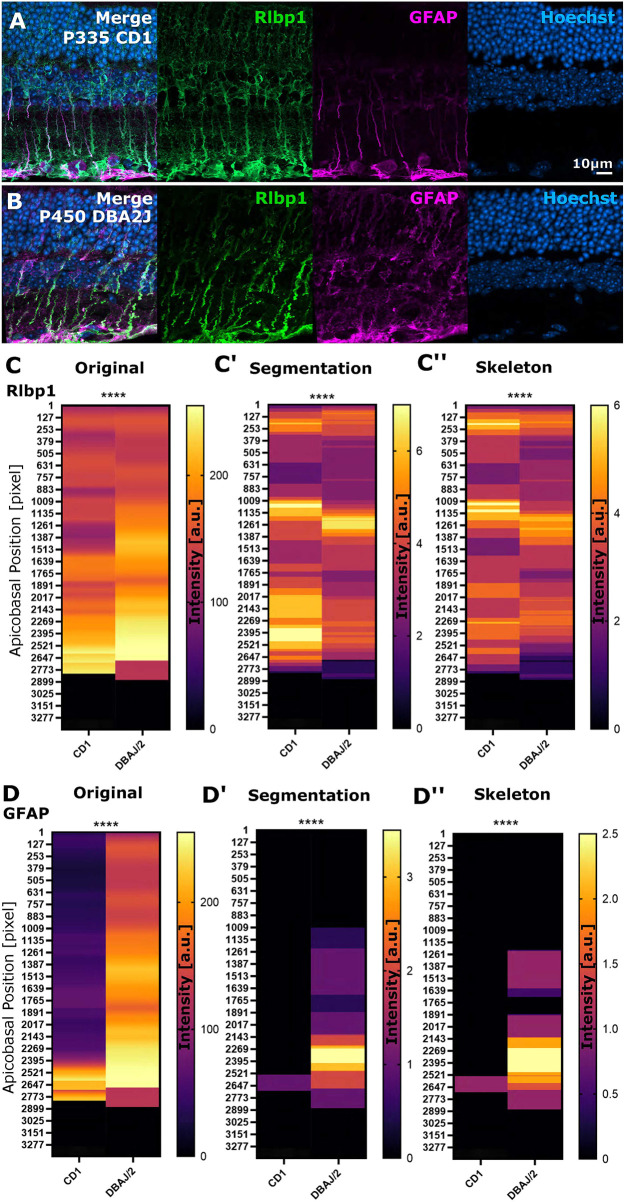
**Apicobasal texture analysis identifies subcellular changes in protein localization in mouse glaucoma models.** (A,B) Micrographs of stainings in controls and spontaneously glaucomatous DBA/2/J mice (Rlbp1 labels MG, GFAP labels MG reactivity, DAPI labels cell nuclei). (C-C″) Apicobasal texture analysis of Rlbp1 using the zonationTool in original, segmented and skeletonized data showed significant structural changes in glaucoma (original ****P<0.0001; segmentation ****P<0.0001; skeleton ****P<0.0001). (D-D″) Apicobasal texture analysis of GFAP using the zonationTool in original, segmented and skeletonized data showed significant subcellular expression changes in glaucoma models (original ****P<0.0001; segmentation ****P<0.0001; skeleton ****P<0.0001). Mann–Whitney *U*-test; *n*=7 stacks from 3 mice each. a.u., arbitrary unit.

So far, we applied GliaMorph to transgenic reporter lines labelling MG in the zebrafish retina. However, many studies in which MG morphology is of interest (e.g. in diseased tissues or drug treatments) may use other models, such as mice, and a cell-specific fluorescent transgenic reporter is not always available. Thus, we tested GliaMorph on the retina of another species (i.e. mice) in which MG are visualized with antibody staining. To assess whether biologically relevant data could be extracted, we collected retinas from approximately 1-year-old CD1 controls and DBA/2J mice, which develop glaucoma-like phenotypes and exhibit gliosis (Turner et al., 2017). We used an Rlbp1 (also known as Cralbp) antibody to label entire MG and a GFAP antibody to detect gliosis and pathology (Fig. 9A,B). Subsequent to establishing similarity with the subregionTool and splitting channels with the splitChannelsTool, we applied the zonationTool to analyse apicobasal distributions. This showed changes in Rlbp1, suggesting that cell morphology and subcellular arrangements are changed in this glaucoma model (Fig. 9C-C″; original *P*<0.0001; segmentation *P*<0.0001; skeleton *P*<0.0001). Additionally, we found GFAP to be upregulated and distributed to a more apical area in glaucoma mice in comparison with controls (Fig. 9D-D″; original *P*<0.0001; segmentation *P*<0.0001; skeleton *P*<0.0001). Volume, branching and size quantifications using the GliaMorph suite showed no statistically significant difference (Fig. S7). Together, this shows that GliaMorph can be used with images from retinas of different species and in combination with antibody staining to observe and quantify MG phenotypes.

### Workflow integration

The GliaMorph toolkit allows the workflow to be individualized for experimental needs based on its modular construction (Fig. 1). Batch processing allows processing of whole experimental folders, increasing throughput and automation. Implementation in the Fiji framework allows cross-platform and licence-free applicability. Implementing codes as macros with graphical user interfaces allows direct use and alteration of code, even by users without any coding experience. This is supported by the online availability of code (https://github.com/ElisabethKugler/GliaMorph), example data (Zenodo: 10.5281/zenodo.5747597), YouTube tutorials (https://www.youtube.com/watch?v=aPFBZZIS7Tg&list=PLaAjG7r5mqQnmkPdktLJqbxotoRfyY72k), as well as a step-by-step user guide Kugler et al. (2023).

## DISCUSSION

### GliaMorph allows robust quantification of MG morphology in the vertebrate retina

Quantifying the morphology of glia is challenging owing to their highly branched and complex morphologies. Generally, quantification of glia cells relies on measurements of cell number (e.g. counting nuclei) or manual cell tracing. However, this provides little detail of the morphology and can be time consuming or prone to human error. Quantifying morphology is crucial because the cell shape facilitates close contacts with retinal neurons, synapses and blood vessels. For example, morphological changes in some glial cells, such as astrocytes, correlates with neuronal alterations (Stogsdill et al., 2017), leading to the potential for subtle glial morphological shifts underlying neuronal dysfunction, neural degeneration and ultimately vision loss (Bringmann et al., 2006; Bringmann and Wiedemann, 2012; Vecino et al., 2016). Our data shows that the transgenic membrane marker increased morphological data points compared with the cytosolic marker in developing MG. However, the global population glial measurements with membrane-tagged fluorophores became obscured because of densely labelled regions of the retina and limitations in the segmentation and skeletonization using GliaMorph. Analysis of developmental changes of MG showed an increase of most measured features, and we show that if global changes are too subtle to be depicted with the quantificationTool, local analysis with the zonationTool can detect MG morphological elaboration at each of the apicobasal domains. To gain additional insights into individual MG morphology, we used single-cell transgenic labelling, in which the elaboration of MG cells at precise domains is most apparent. This demonstrates the importance of considering which cellular labels are used to visualize and quantify glial morphology. For example, even though glia shape serves as a readout of maturity (MacDonald et al., 2017) or cell damage (Halford et al., 2017), plasma membranes can suffer disruption in these conditions, interfering with morphological analysis (Halford et al., 2017).

The cellular and molecular mechanisms regulating the elaboration of MG precisely at each of the five domains in the retina remain poorly understood. GliaMorph will provide a robust computational pipeline to analyse and quantify MG shape in genetic mutants or knockdown of candidate genes (Escartin et al., 2021; Charlton-Perkins et al., 2019). Importantly, as techniques develop, future work will also be looking at integrating multimodal data, such as shape analysis, calcium data for cell function, and overall visual behaviours. By applying apicobasal texture analysis to a mouse disease model, we show that there is an overall change in expression domains in MG in a glaucoma model. This is particularly pertinent to the pathogenesis of disease, whereby subtle glial cell changes may serve as important hallmarks of neurodegeneration, even potentially driving pathogenesis. In many instances, transgenic or single-cell labels are not possible. Here, we have shown that using antibodies on a mouse model of retinal disease and the zonationTool we can clearly identify subcellular expressions changes of a common glial pathology marker (e.g. GFAP). Thus, the zonationTool is a powerful tool for rapid subcellular morphological analysis in any retinal tissue that can be labelled with cell-specific florescent transgenic reporters (e.g. zebrafish) or with glia-specific antibodies (e.g. mouse or human). This global level analysis can be expanded upon with the segmentationTool and quantificationTool to quantify precise cellular features.

### The importance of imaging data quality and standardization

Standardized image analysis approaches, especially those that facilitate the reliable comparison of images across different samples and labs, are crucial for the rapidly advancing imaging paradigms and large datasets acquired in cell and developmental biology. As more and more image data are standardized, and protocols for depositing raw image data are developed, approaches like GliaMorph will become more commonplace. Here, we present a comprehensive data analysis workflow to assess 3D MG morphology. We show that in-depth data understanding is crucial to analysing data in 3D. We performed benchmarking and troubleshooting using a variety of experimental approaches to identify key parameters that must be optimal/optimized for workflow validity. Although we included sections on data understanding and deconvolution, these need to be newly assessed when working with data other than those studied here. As the field of deconvolution is complex, we here only suggest the two routes: (1) deconvolution of confocal data with existing Fiji plugins integrated into the deconvolutionTool and (2) microscope programs designed specifically for their data (e.g. processing Airyscan-acquired data with Zeiss packages). Again, it is important to spend time on data exploration and examination to understand the data and what features need to be considered [e.g. highly different contrast-to-noise ratio (CNR) between different subcellular regions] (see the Olympus ‘Introduction to Deconvolution, https://www.olympus-lifescience.com/en/microscope-resource/primer/digitalimaging/deconvolution/deconintro/; Huang and Murphy, 2004). Generally, the better the data, the better the data analysis output.

MG have a computationally challenging apicobasal arrangement, which translates to five subcellular domains that are biologically and computationally highly distinctive. In addition to subcellular difference, local anatomical differences can impact data analysis. Thus, to obtain comparable datasets, image acquisition should be performed at standardized positions. Here, we acquired data in the ventro-temporal zone of the right eye [dorsally to the area temporalis, known as strike zone (Zimmermann et al., 2018), or high-acuity area]. Standardization procedures might differ for samples at other ages, visualization techniques (e.g. different transgenics, antibodies, microscopes, etc.), or species. We also highlighted the challenges in *z*-axis signal decay in confocal microscopy, and suggested that the appropriate deconvolution is a requirement for fluorescence microscopy, which is in line with previous work (Korobchevskaya et al., 2017; Tröger et al., 2020). However, particularly for antibody staining, penetration depth in sample preparation is a limiting factor for image quality. Again, these observations highlight that high input data quality and appropriate image pre-processing are pivotal for quantitative image analysis. As data are often acquired at different orientations, we achieved data comparability by employing semi-automatic rotation and 3D subregion selection. Standardization allowed for dimensionality reduction using the zonationTool to create 1D vectors from 3D data, enabling intuitive and quantitative insights into apical-basal polarity data. Lastly, employing standardized data, we used fibre-orientation assessment to analyse vertical-to-horizontal structures in our data. Together, high-quality data, data understanding, and establishing image comparability allowed for data analysis and quantification on various levels. GliaMorph allows for multi-dimensional glia analysis, enabling image-level as well as subcellular assessments that are robust, easy to use and adaptable.

### Robustness of application

An aim for data analysis approaches should be robustness across users and data. Using acquisition standardization and automatic analysis ensures comparability between age-matched samples. This is exemplified when acquiring two datasets by two independent investigators and comparing MG volume and skeleton as readouts. This showed neither a significant difference, nor bias, for both measured parameters. Thus, even if data are acquired in different samples and by different people, age-matched analysis is possible (Fig. S8A,B; volume *P*=0.9211; skeleton *P*=0.8460). Similarly, analysis of the same dataset, but by different experimenters does not bias/change the parameters analysed by GliaMorph (Fig. S8C,D; volume *P*=0.1934; skeleton *P*=0.7363). This shows that the GliaMorph pipeline (Fig. 1) is a robust and reliable method to quantify MG morphology, which will facilitate the direct comparison of data between multiple users and laboratories. Additionally, we here presented data acquired by markedly different approaches, varying experimenters, transgenic lines or antibody staining, whole-mount intact tissues or cryosections, different microscopes and zebrafish or mouse tissues, and were able to perform multi-dimensional data analysis and detect subtle differences that might be otherwise overlooked using visual or manual assessments.

### Applicability to MG in other species and other cell types

GliaMorph can detect features including positioning of nuclei, subcellular features along the apicobasal axis, general cell morphology features, including branch points, cell body size, and skeletal shape. Similar comparisons can be carried out in other vertebrate models to determine whether the MG cells undergo a stereotypic morphological elaboration programme in the developing retina. As GliaMorph is modular, it can be adapted for other tools or techniques to visualize glial cells. This could be other transgenic reporter lines or antibody staining, but also data from different species and potentially other cell types, including neurons or other complex cells in the CNS. For example, the subregionTool provides a way to select similar ROIs of different images and analyse global level structural changes across the FOV. This can be applicable for imaging data from any tissue, not just in the CNS, where changes in fluorescence intensity across the FOV provides meaningful biological data. Another consideration is the analysis of whole-mount intact tissues versus cryosectioned tissue data, as the latter tends to be impacted by tissue integrity. Together, all steps, except image segmentation, of the GliaMorph toolkit are directly applicable to data other than that presented here. However, application to other data needs to come with the caveat that data often differ in sample preparation (e.g. different antigen retrievals, bleaching or fixation) and image properties (e.g. autofluorescence). As mentioned, the main bottleneck is the segmentationTool (Table 3), which will require optimization for any data analysed other than that presented here. Most of the developed tools will be independent of input data properties, such as microscope type or cell features (e.g. radial glial cells have a similar radial structure to MG; see Table 3 for more detail). However, for all analyses, good image quality and standardization are key for meaningful quantifications.

**Table 3.**
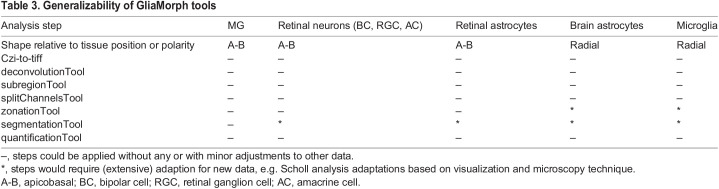
Generalizability of GliaMorph tools

## Conclusion

We developed a comprehensive image understanding and analysis workflow, which is modular and open source in application. Our presented work is an important benchmark study on how to understand and analyse retinal MG data.

## MATERIALS AND METHODS

### Zebrafish handling and husbandry

Experiments performed at UCL conformed to UK Home Office regulations and were performed under Home Office Project Licence PP2133797 held by R.B.M. Maintenance of adult zebrafish in the fish facilities was conducted in Aquaneering tanks with a density of five animals per litre according to previously described husbandry standard protocols at 28°C with a 14:10 h light:dark cycle (Westerfield, 1993; Aleström et al., 2019). Embryos, obtained from controlled pair or group mating, were incubated in E3 buffer (5 mM NaCl, 0.17 mM KCl, 0.33 mM CaCl_2_, 0.33 mM MgSO_4_) with/without Methylene Blue and 0.0045% 1-phenyl 2-thiourea (Karlsson et al., 2001) applied between 6 and 24 hpf and refreshed at a minimum of every 24 h.

### Zebrafish strains

To visualize MG, the following transgenics were used: *Tg(tp1bglob:VenusPest)^s940^* (Ninov et al., 2012), *Tg(CSL:mCherry)^jh11^* (also known as *Tg(tp1bglob:hmgb1-mCherry)^jh11^*; Parsons et al., 2009). Retinal ganglion cells, photoreceptors, amacrine cells and horizontal cells were visualized with *Tg(ath5:gapRFP)^cu2^* [also known as *Tg(atoh7:gap43-mRFP1)^cu2^*; Zolessi et al., 2006]. Bipolar cells were visualized with *Tg(vsx1:GFP)^nns5^* (Kimura et al., 2008). Amacrine and horizontal cells were visualized with *Tg(ptf1a:dsRed)^ia6^* (Jusuf et al., 2012) and *Tg(ptf1a:cytGFP)* (Godinho et al., 2005)*.*

### Mouse animal care

All animal husbandry was conducted according to the guidelines of the Canadian Council on Animal Care, using University of Ottawa ethical protocols OHRI-2856 and OHRI-3499. Animals were housed under specific pathogen-free conditions in standard isolation cages with enrichments. Animals were provided with food and water *ad libitum*. CD1 mice were obtained from Charles River Laboratories, and DBA2/J mice were obtained from Jackson Laboratories. Animals of both sexes, aged 335 days to 1 year, were used in this study.

### Construct generation

The expression constructs *pTol2-tp1bglob:eGFP-CAAX;cmlc2:eGFP* and *pTol2-tp1bglob:mCherry-CAAX;cmlc2:*eGFP were generated using recombination reactions with LR-clonase II (Invitrogen) as standard in Multisite Gateway Technology (Kwan et al., 2007). These constructs were generated by combining purified plasmid DNA (QIAGEN QIAprep Spin Miniprep Kit, 27104) for the p5E-tp1 (Addgene plasmid #73585, deposited by Nathan Lawson; Quillien et al., 2014), pME-mCherry-CAAX [a gift from Yi Feng's group (Kwan et al., 2007)] or pME-eGFP-CAAX with the p3E-polyA into the pDestTol2CG2 vectors (Kwan et al., 2007) from the zebrafish Tol2Kit. The purified DNA for these constructs (25 ng/µl) were microinjected into one-cell-stage zebrafish embryos with Tol2 mRNA (25 ng/µl) to generate stable transgenic lines for single-cell MG analysis using low-level concentration injection (13 ng/µl).

### Microinjections and generation of stable lines

Microinjections were performed using a borosilicate glass capillary needle (World Precision Instruments, TW100F-4) connected to a Pneumatic Picopump injector (World Precision Instruments). To generate mosaic labelling of MG, 6.5 pg of *pTol2-tp1bglob:eGFP-CAAX;cmlc2:eGFP* or *pTol2-tp1bglob:mCherry-CAAX;cmlc2:GFP* was co-injected with 25 pg of purified capped Tol2 transposase mRNA in a volume of 0.5 nl, into one-cell-stage zebrafish embryos. To establish the *Tg(tp1bglob:eGFP-CAAX)* stable line, injected embryos were screened for GFP expression in the heart at 48-72 hpf. The F1 generation was screened for eGFP-CAAX expression to identify founder fish with germline integration and stable transmission to offspring. Positively identified F1 larvae were grown to adulthood and a stable transgenic line was established based on F2 generation with the strongest and most pervasive expression.

### Fixation

Embryos were fixed with 4% paraformaldehyde (Thermo Fisher Scientific, 28908) in PBS for 2-4 h at room temperature. Dehydration was performed by consecutive 5-min washes with 25%, 50%, 75% methanol in PBS and twice with 100% methanol. Samples were stored at −20°C in methanol. Rehydration was performed in the reverse order and followed by three 5-min washes in PBS. Fixation was conducted at 24, 48, 60, 72, 96 and 120 hpf (staging based on anatomical features).

### Zebrafish immunohistochemistry

*Tg(TP1bglob:VenusPEST; -5.5 ptf1a:DsRed)* embryos were fixed in 4% paraformaldehyde overnight at 4°C. Immunostaining was carried out on whole embryos as previously described (Inoue and Wittbrodt, 2011), with heating in 150 mM Tris-HCl pH 9.0 at 70°C and subsequent −20°C acetone incubation. Embryos were then incubated with primary antibodies [mouse anti-Gfap (1:100; zrf1, ZIRC), rabbit anti-Rlbp1 (1:200; 15356-1-AP, Proteintech), mouse anti-glutamine synthetase (1:200; mab302, Merck)], followed with secondary antibodies [goat anti-mouse IgG Alexa Fluor^®^ 647 (A21235, Thermo Fisher Scientific) and goat anti-rabbit IgG Alexa Fluor^®^ 488 (A11008, Thermo Fisher Scientific)]. After immunohistochemistry, embryos were incubated in 1 μg/ml of 4′,6-diamidino-2-2 phenylindole (DAPI; Roche, 10236276001; stock concentration: 1 mg/ml; working concentration: 1 µg/ml in PBS) overnight at 4°C and washed in PBS once prior to imaging.

### Mouse immunohistochemistry

Immunohistochemistry was performed essentially as described previously (Mattar et al., 2015). Briefly, eyes were harvested from mice euthanized by CO_2_ overexposure before cervical dislocation, and the corneas and lenses were removed. Eye cups were fixed with 4% paraformaldehyde in PBS for 10 min at room temperature. After cryoprotection overnight in 20% sucrose in PBS, retinas were cut into 18 µm sections using a Leica CM1900 cryostat. Primary antibodies were anti-Cralbp (Rlbp1; Thermo Fisher Scientific, MA1-813) and anti-Gfap (Millipore Sigma, AB5804).

### Image acquisition

Zebrafish images were acquired on a Zeiss LSM 900 with Airyscan 2 using a 40× water-immersion LD C-Apochromat [numerical aperture (NA) 1.1] or 63× oil-immersion Plan Apochromat (NA 1.4) with laser lines 405, 488, 561, 640 nm. Embedding was performed using glass-bottom dishes and 1% low-melting-point agarose (Sigma-Aldrich, A9414), which was covered following solidification with E3.

Mouse data were acquired on a Zeiss LSM900 with Airyscan 2 using a Plan-Apochromat 63×/1.40 Oil DIC f/ELYRA.

Sampling frequency was assessed for *Tg(TP1bglob:VenusPest)^s940^* using the Nyquist sampling rules (https://svi.nl/NyquistCalculator; Eqns 1-3):
(1)

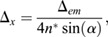

(2)

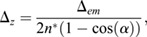

(3)

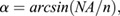
For calculations, an Array Detector was used as microscope type, with the following parameters/settings: numerical aperture, 1.3; excitation wavelength, 488; emission wavelength, 520; number of excitation photons, 1; lens immersion refractive index, 1.338. Zeiss AiryScan was used as Array Detector model. This resulted in a Nyquist sampling of: *x*, 46 nm; *y*, 46 nm; *z*, 119 nm.

### Image analysis

Images were analysed using open-source software Fiji (Schindelin et al., 2012). Samples were excluded from analysis if image quality did not allow for reliable quantification (e.g. significant intra-plane movement, low CNR due to transgenic reporter weakness, unviable cell or animal health). Animals were allocated to treatment groups randomly without selection. Imaging and data analysis were performed unblinded to treatment allocation, often because the effect of treatment was easily deduced from the appearance of the micrograph. To overcome subjective bias, objective automized image analysis was applied where possible.

### Image understanding

Manual measurements of retina size and MG cell bodies were performed using a line ROI (for details, see workflow document in Kugler et al., 2023). Manual measurements of intensity distributions were performed using a line ROI and plotting intensity values from maximum intensity projections (MIPs).

CNR (Eqn 4) and signal-to-noise ratio (SNR; Eqn 5) were quantified by the placement of 3-5 μm circular ROI at slices of interest at the position of the cell body, protrusion and endfoot for mean signal measurement, non-signal ROIs were placed inside the retina without cellular signal, and background ROI was placed outside the retina:
(4)

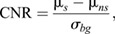

(5)

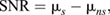
where µ_*s*_ is the mean signal, µ_*ns*_ is the mean non-signal and σ_*bg*_ is the standard deviation of the background.

### Image pre-processing

#### subregionTool

This tool rotates images to align them along the *y*-axis, based on manual line ROI selection (start point at the MG endfeet and end point apically). All images in the study are represented with apical being the top of the panel and basal at the bottom. Following the rotation, a bounding box was established with a 60 μm default width (*x*-axis) and box length was defined by the length of the manual line ROI with an extension of sigma (default 10 μm) to allow for the inclusion of the underlying vasculature and consideration of retinal curvature. The position of the bounding box was determined by the endpoint of the line ROI following rotation with the following potential cases: (1) *xy*-defined box fits into the image; (2) box overfits on the right, meaning the box will start at point xT-bT, with xT being the image width and bT being the box width; (3) box overfits on the right, meaning the box will start at *x*=0; (4) box overfits on the bottom, meaning the output image height will be the length of the manual line ROI (error message ‘File X sigma could not be attached’, with X being the file name). Next, the image was cropped in *xy* dimensions to this size saved, and the stack was reduced to the default 10 μm thickness. Should the stack not be thick enough an error message is produced: ‘File X not enough slices for substack’, with X being the file name.

#### 90DegreeRotation

This tool rotates images 90° to the left or right before application of the subregionTool and is required when images are rectangular rather than cubic, potentially resulting in artificial cropping.

N-tuple multi-labelling was established by image merging and false-colour look-up table (LUT) application following the application of the subregionTool to the respective embryos.

#### zonationTool

Following 8-bit conversion, MIPs (*xy* direction) were produced, images were transformed 90° from the left (Image>stack>reslice; Fig. 3D) and additional MIPs (*z* direction) produced. For intensity plotting, image width can be scaled to 1920 voxels using bilinear interpolation to allow for comparability between different images. A line ROI was automatically placed starting from pixel 0 (left-most) to the image width, and intensity profiles were measured and saved (one output file per input folder). One-voxel wise representation was assigned LUT Fire based on intensity and saved. Derived measurements were the total image height (*I_N_*) and retina height (*R_N_*), whereas retina height is based on sigma added with the subregionTool.

#### Airyscan processing

Airyscan processing was conducted (independently of the transgenic used or staining) with Zeiss Zen Blue Software using 3D standard unless otherwise indicated in the text. Briefly, the processing was performed individually on images acquired with the 32 detectors, using a non-iterative linear Wiener deconvolution algorithm (Huff, 2015; see also Zeiss LSM 900 – Basic User Notes Airyscan).

As the processing is included in the Zeiss software system, there was no requirement to include additional steps into GliaMorph. However, we wanted to use this informed approach to establish the ideal processing before converting to GliaMorph. First, we examined the impact of Airyscan processing using the settings 2D standard, 3D standard and 3D high (Fig. S4A-D). As expected, this showed 3D deconvolution to outperform 2D deconvolution, as assessed per reduced background (white arrowheads), and high deconvolution to result in increased structured noise and grains (black arrowheads). We next assessed this quantitatively using CNR measurements. This showed that 3D deconvolution indeed outperformed 2D processing and that reduction of the 1 μm *z*-stack step size to 0.19 μm increased the CNR drastically (Fig. S4E,F). As such, these parameters are feasible and ideal for MG morphological data in the zebrafish retina.

#### deconvolutionTool

##### PSF modelling

Theoretical PSF was modelled using analytical derivation based on Fraunhofer diffraction using the ‘Diffraction PSF 3D’ plugin of ImageJ (https://imagej.net/Deconvolution; https://www.optinav.info/Diffraction-PSF-3D.htm). The objective NA can be changed freely; the following standard suggestions were chosen: 20× Air NA 0.8, 40× Water NA 1.1, 63× Oil NA 1.4. Fluorophore wavelengths were entered manually for up to four channels.

##### Confocal PSF deconvolution

Deconvolution of the image *y* was performed with the plugin DeconvolutionLab2 (Sage et al., 2017) (http://bigwww.epfl.ch/deconvolution/deconvolutionlab2/):
(6)

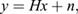
where *y* is the data, *H* is the PSF matrix, *x* is the image and *n* is the added noise component.

The following deconvolution algorithms were examined:

###### (1) Regularized Inverse Filter (Ali, 2011):


(7)

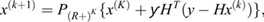
where *L* is the discretization of the Laplacian operator, *T* denotes the adjoint of *L* and *H*, and λ is the regularization factor (set to 1.000E−18).

###### (2) Landweber (Landweber, 1951) iterative deconvolution:


(8)

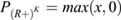
where 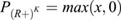 is the component-wise projection, the number of iterations *M_iter_* (set to 15), and step-size parameter *ƴ* (set to 1.5).

###### (3) Fast iterative shrinkage thresholding (Beck and Teboulle, 2009) with the cost function:


(9)

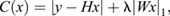
where *W* represents a wavelet transform and **λ** is the regularization factor (set to 1.000E−18).

###### (4) Bounded-variables least squares, also known as Spark–Parker algorithm (Stark and Parker, 1995), minimizing a least-squares cost function (variables constraint):


(10)

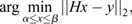
where *y* is the vector of response variables, **||**.**||_2_** denotes the Euclidean norm, and α≤*x*≤β denote the upper and lower bounds, respectively.

###### (5) Richardson–Lucy (Richardson, 1972; Lucy, 1974) with the assumption of Poisson noise and the cost function:


(11)

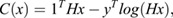
with *M_iter_* (set to 1 or 5).

To assess image quality quantitatively, CNR was quantified. To account for subcellular differences (Fig. S1A), CNR was measured in several MG subdomains (MG cell bodies, protrusions and endfeet). When examining the impact of Richardson–Lucy RL iteration numbers visually, one iteration was found to preserve MG IPL protrusions (Fig. S3, unfilled arrowhead) but not fully remove retina auto-fluorescence/background (green arrowhead), whereas five iterations did not fully preserve IPL protrusions but removed the observed background. This was confirmed when examining image intensities (Fig. S3H). When quantifying CNR levels, five iterations delivered higher CNR outcomes than one iteration (Fig. S3I-K). However, considering the loss of detail in IPL protrusions with five iterations, one iteration was used subsequently.

##### User choice summary

The above was then integrated into the deconvolutionTool with user choices as follows: single-/multi-channel, selection of fluorophores by manual input of wavelength (nm) for up to four channels, NA input, and non-/existing PSF file.

### Segmentation

#### Zebrafish *Tg(TP1bglob:VenusPest)^s940^* and *Tg(CSL:mCherry)^jh11^*

Bleach correction was performed using the ‘Simple Ratio Method’ with background 0 (Miura, 2020). Subsequently, images were converted to 8-bit to allow for the following steps. Smoothing was performed using a 3D median filter with a radius of 2 voxels (Lim, 1990). Segmentation was conducted after the selection of the plane at the middle of the stack with Otsu thresholding (Otsu, 1979) (histogram-derived):
(12)

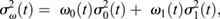
where *ω*_0_ and *ω*_1_ are the probabilities of the two classes to be separated with the threshold (*t*), calculated from the histogram, and *σ*_0_ and *σ*_1_ are the class variances, respectively. For post-processing, 3D median filtering with a radius of 2 voxels and binarization were conducted for surface smoothing. Additionally, MorpholibJ ‘Keep Largest Region’ from the IJPB-plugins was used (Legland et al., 2016).

#### Zebrafish *Tg(TP1bglob: TP1bglob:eGFP-CAAX)^u911^*

Prior to segmentation, the ‘3D Edge and Symmetry Filter’ was applied (settings: ‘alpha=0.500 compute_symmetry radius=10 normalization=10 scaling=2 improved’) (Ollion et al., 2013). Segmentation was carried out as described above, with post-processing as follows: 3D hole filling (Ollion et al., 2013) instead of surface smoothing.

### Quantification

All quantifications were performed using segmented 3D stacks as input.

To quantify the number of MG (*N_N_*), three approaches were tested: (1) automatic ROI selection and MG counting, (2) semi-automatic ROI selection and automatic MG counting, and (3) manual measurements (taken as gold standard). Briefly, to select ROI automatically, the zonationTool was applied to the segmented 3D stacks to extract the MG cell body position per image, whereas for the semi-automatic approach, one rectangular ROI was drawn per experimental group (this assumes that animals are age matched and comparable within the group). For automatic cell counting, stacks were cropped to ROI size in *xy* dimensions. Then ‘Distance Transform Watershed 3D’ (Legland et al., 2016) was applied to separate cell bodies, followed by ‘3D Simple Segmentation’ (Ollion et al., 2013) for binarization, and the BoneJ ‘Particle Analyser’ (Doube et al., 2010) to quantify the number of cells.

MG volume (*V_N_*) was quantified as the number of object voxels (zero value) in histogram, multiplied by voxel size; volume coverage [*VC_N_* (%)] was measured as percentage of image voxels covered with MG object voxels.

Surface measurements (*S_N_*) were derived as surface/edge voxels following segmentation, and, because of uncertainty in orientation (i.e. face, edge, or vertices) and normalization (i.e. voxel size differences between datasets), were given as a volume instead of area or number. Lastly, the surface-to-volume ratio (*S:V_N_*) was derived as a ratio.

Centreline extraction was performed in segmented images using the Fiji ‘Skeletonize 2D/3D’ plugin, based on 3D thinning (Lee et al., 1994). Centreline voxels (zero-valued in images) were quantified for total network length (*L_N_*) analysis by quantification of object voxels (zero value) in histogram.

The ‘Analyse Skeleton’ plugin in Fiji (Analyse>Skeleton>Analyse Skeleton 2D/3D; Arganda-Carreras et al., 2010) was used to identify and measure the number of junctions (*J_N_*), number of endpoints (*EP_N_*) and average branch length (*BL_N_*) (Analyse>Skeleton>Summarize Skeleton).

Euclidean distance maps (*EDM_n_*) of object voxel distance to the nearest background voxel were produced from binary segmented images using the Fiji plugin ‘Distance Map 3D’, which calculates distance in 3D Euclidean space (Eqn 10; Process>Binary>Distance Map in 3D; Borgefors, 1996). To quantify thickness (*T_N_*), EDMs were multiplied with extracted skeletons, resulting in a 1D representation of vessel radii as represented by intensity of voxels (see Kugler et al., 2022).

### Fourier transformation analysis

To obtain information on MG alignment, alignment by Fourier transform (AFT), a previously published open-source tool for evaluating the alignment of fibrillar structures across different length scales, was used (Marcotti et al., 2021). This tool allows for local evaluation of alignment by exploiting the representation of small tiles of the image in the Fourier space. We achieved alignment analysis of MG by building on the AFT workflow as follows. We used (1) the subregionTool to produce equally sized tiff MIPs, (2) CheckImageSize.ijm to identify the largest image, and (3) NormalizeImageSizes.ijm to make data comparable by top-to-top alignment and attachment of black voxels to achieve image height similarity, using the largest image as standard; this step also includes an image enhancement step. We then applied (4) AFT only to non-black/non-background voxels [parameters: window size 100 pixels, overlap 50%, neighbour radius three vectors (i.e. the neighbourhood of seven vectors); save output images – yes; apply filtering – yes; masking – 0, ignore blank spaces – 1, ignore isotropic regions – 0; mean background 5], and we produced (5) line-based averages along the apicobasal axis (i.e. *x* averages iterated over the *y*-axis).

#### Data representation

To visualize data, MIPs were generated; intensity inversion was applied as appropriate to give the clearest rendering of relevant structures. Depth coding was performed with the ‘Temporal-Color Coder’ by Kota Miura (https://imagej.net/plugins/temporal-color-code).

### Statistical analysis

Normality of data was tested using D'Agostino-Pearson omnibus test. Statistical analysis of normally distributed data was performed using a one-way ANOVA to compare multiple groups or two-tailed, unpaired Student's *t*-test to compare two groups. Non-normally distributed data were analysed with a Kruskal–Wallis test to compare multiple groups or Mann–Whitney test to compare two groups. Analysis was performed in GraphPad Prism Version 9 (GraphPad Software). *P*-values are indicated as follows: *P<0.05, **P<0.01, ***P<0.001, ****P<0.0001. All data were acquired as experimental repeats rather than replicates. Based on the mean/s.d. of the data in the examined groups in the assays used, post-hoc power calculations have shown that these assays have at least 80% power to detect an effect size of 30% difference between groups when group sizes are 12/group (alpha=0.05). Image representation was performed using Inkscape (https://www.inkscape.org).

## Supplementary Material

10.1242/develop.201008_sup1 Supplementary information

## Data Availability

Code used in this study is available at https://github.com/ElisabethKugler/GliaMorph. Example data for the #GliaMorph Protocol are available at Zenodo under accession number 5747597, Minimum Example Data under accession number 5735442, and Double-transgenic Müller Glia Data at 3 dpf and 5 dpf zebrafish under accession number 5938758.
