# Supplementary material for: GliaMorph: a modular image analysis toolkit to quantify Müller glial cell morphology
Source: Development. 2023 Feb 2;150(3):dev201008. doi: 10.1242/dev.201008 (PMC10110500; doi:10.1242/dev.201008)
Supplement: Supplementary file 1 [file develop-150-201008_review_history.pdf]

# GliaMorph: a modular image analysis toolkit to quantify Müller glial cell morphology

Elisabeth Kugler, Isabel Bravo, Xhuljana Durmishi, Stefania Marcotti, Sara Beqiri, Alicia Carrington, Brian Stramer, Pierre Mattar and Ryan B. MacDonald
DOI: 10.1242/dev.201008

**Editor**: James Briscoe

**Review timeline**

| | |
|---|---|
| Original submission: | 8 June 2022 |
| Editorial decision: | 29 July 2022 |
| First revision received: | 1 December 2022 |
| Accepted: | 3 January 2023 |

---

**Original submission**

First decision letter

MS ID#: DEVELOP/2022/201008

MS TITLE: GliaMorph: A modular image analysis toolkit to quantify Müller glial cell morphology

AUTHORS: Elisabeth C Kugler, Isabel Bravo, Xhuljana Durmishi, Stefania Marcotti, Sara Beqiri, Alicia Carrington, Brian C Stramer, Pierre Mattar, and Ryan B MacDonald

I have now received all the referees' reports on the above manuscript, and have reached a decision. The referees' comments are appended below, or you can access them online: please go to BenchPressand click on the 'Manuscripts with Decisions' queue in the Author Area.

As you will see, the referees express interest in your work, and suggest it has the potential to provide a useful resource/technique to the community, but the referees have some significant criticisms and recommend a substantial revision of your manuscript before we can consider publication. I agree with Referee 1 that revising the study to focus on the insights gained from the implementation of GliaMorph would strengthen the paper. I also agree that, without additional controls and analysis, the conclusions from the Crispant experiments are too preliminary. Streamlining the paper to focus on the implementation of the method would make the work more accessible. However, if this is the approach you adopt to the revision, it will be important that the study remains distinct from the Current Protocols manuscript you are also preparing.

If you are able to revise the manuscript along the lines suggested, which may involve further experiments, I will be happy receive a revised version of the manuscript. Your revised paper will be re-reviewed by one or more of the original referees, and acceptance of your manuscript will depend on your addressing satisfactorily the reviewers' major concerns. Please also note that Development will normally permit only one round of major revision. If it would be helpful, you are welcome to contact us to discuss your revision in greater detail. Please send us a point-by-point response indicating your plans for addressing the referee’s comments, and we will look over this and provide further guidance.

Please attend to all of the reviewers' comments and ensure that you clearly highlight all changes made in the revised manuscript. Please avoid using 'Tracked changes' in Word files as these are lost in PDF conversion. I should be grateful if you would also provide a point-by-point response detailing

© 2023. Published by The Company of Biologists under the terms of the Creative Commons Attribution License (https://creativecommons.org/licenses/by/4.0/).

how you have dealt with the points raised by the reviewers in the 'Response to Reviewers' box. If you do not agree with any of their criticisms or suggestions please explain clearly why this is so.

Reviewer 1

*Advance summary and potential significance to field*

This study describes GliaMorph, a semi-automated, non-deep learning approach to quantifying morphological parameters of Müller glia in the vertebrate retina called. It is a companion to a detailed methods paper submitted elsewhere (and supplied in the supplemental files here) and accompanied by a series of YouTube video tutorials for implementing GliaMorph.

Standardized image analysis approaches that include steps such as feature extraction and the ability to reliably compare images across samples/labs and conditions is a current and growing field within cell and developmental biology.

As more and more image data is standardized and protocols for depositing raw image data are developed (and required) approaches like the one described in this paper will become commonplace. This manuscript includes information about the specific approaches the researchers took to build GliaMorph. The authors give an overview of the steps required to establish and then apply GliaMorph showing that the approach can detect features including positioning of nuclei and other cellular features along the apicobasal axis, general cell morphology features including branch points, cell body size, and skeletal shape. In general, GliaMorph appears to be a useful approach for reproducible image analysis of M. The authors also apply GliaMorph to other samples to test whether it can detect differences in Müller glia cells across different conditions.

Because this manuscript describes a new tool and validates it, but does not contribute a significant biological conceptual advance, I would suggest that this paper could be considered as a Resource/Technique rather than a Research article as the novelty is in the method and approach rather than the biological findings.

*Comments for the author*

I enjoyed reading this manuscript and previewing GliaMorph. Below are a number of suggestions for improving the readability of the manuscript as well as ensuring the accuracy of the study.

1. Overall organization of the paper could be improved with a number of changes including considering how to organize the figures so that they are discussed in the order in which they appear in the manuscript and by moving figure 10 closer to the beginning of the manuscript (possibly after Figure 1). Moving Figure 10 up will provide a roadmap for the reader, using it to set up the flow of the paper which essentially walks through how each part of the workflow was established and then validates the approach by analyzing MG morphology over time and assessing retinal disease mouse tissue sections.

2. Some thoughts about statistics and the plots. It’s been my understanding that the Kruskal-Wallis test is used when you have a ranked variable and a nominal variable. It’s unclear to me which is the ranked variable in the analyses (maybe the developmental stages). The Kruskal-Wallis test is NOT a test of the difference of means or medians, rather the null hypothesis that is tested with the Kruskal-Wallis test is that the *mean ranks* of the groups are the same. The expected mean rank depends on the total number of observations and isn’t as representative of the distribution (and means/medians) of the data. The Mann-Whitney-Wilcoxon test is the non-parametric version of a two-tailed t-test and is similar to the Kruskal-Wallis test. It may or may not be the most appropriate statistic as it is unclear whether the data were tested for the shape of their distribution. The majority of the plots show, I assume, mean and standard deviation, but what exactly is plotted needs to be more clearly indicated in the figure captions.

3. The separation of the Results from much of the Methods section particularly the sections of Image Analysis through Statistical Analysis, does not work so well for this particular manuscript. From my perspective, the Methods for all the steps of GliaMorph are integral to, if not actually part

© 2023. Published by The Company of Biologists under the terms of the Creative Commons Attribution License (https://creativecommons.org/licenses/by/4.0/).

of the Results for this study. This speaks to the larger issue with this manuscript – as it seems that this manuscript is a validation/application of GliaMorph providing a way to more systematically and quantifiably describe the features previously described for Müller Glia. It is my view that with extensive rewriting/revision that streamlines that study and focuses on the insights gained from the implementation of the tool (such as those described in Figures 3-6) could improve the impact of the paper.

4. The analysis of cdh2 crispants should be removed. This part of the manuscript detracts from the overall message of the paper and isn't as robust as the data from mice retinae. I also have some technical concerns with this experiment and those are detailed below.

5. Images are oriented with apical up and basal down which is fine, but in the ophthalmology/clinical fields basal is up and apical is down. Moreover, when embryologists orient images, the convention is typically dorsal as up and ventral down. Maybe a note about the orientation of the images somewhere prominent and/or early in the results would be helpful for all readers.

6. All figure captions need to describe the figures not report results. The running title for each figure caption can summarize the results of the figure but what is in the figure should be described. For example, for the plots with data points, instead of writing that there was or wasn’t a statistically significant difference, describe the axes and the type of plot and what the data points represent.

7. While Müller glia definitely have some features of astrocytes, they also bear resemblance to radial glia, especially in organisms that can regenerate and/or grow continuously.

8. There are many additional useful resources/companions to this manuscript. The YouTube videos for implementation and the written protocol should be prominently cited to improve the likelihood that this tool will be widely used.

Specific comments:

Methods:

Construct generation: need more details/references for the plasmids and Multisite Gateway technology. Did the authors use purified components from their own lab or did they purchase a kit? Does the pME-mCherry-CAAX plasmid have a reference or a way that others could obtain it?

Cdh2 CRISPR/Cas9 injections: How was the efficacy of CRISPR/Cas9 evaluated? What controls were used to confirm specificity?

Fixation: Dehydration was performed by consecutive 5-minute washes with 25%, 50%, 75% Methanol (MeOH) in PBS. Instead of “Dehydration was performed by consecutive 5 minute washes with 25%, 50%, 75% in PBS….”

Immunohistochemistry: DAPI is 4′,6-diamidino-2-2 phenylindole, not 4′,6-diamidino-2- 532 phenylindole. DAPI staining typically follows immunohistochemistry. What was the stock concentration of DAPI? Was it diluted from a stock that was made in DMSO or PBS?

What was the temperature for acetone incubation?

Image Analysis and on:
Line 750 – CNR abbreviation is used before it is defined on line 762 Lines 751-754 seem superfluous since the main point of the paper is the new image analysis method.

Results with a focus on Figures and Tables:
Table 1 needs some footnotes to be more readily understood. For example, I’m assuming that A-B stands for apical to basal, but that needs to be explicit and some explanation about how exactly radial applies to microglia and astrocytes.
What is the significance of the two colors?

© 2023. Published by The Company of Biologists under the terms of the Creative Commons Attribution License (https://creativecommons.org/licenses/by/4.0/).

Table 2 seems out of place in the methods; it seems more like part of the Results since it is describing the features of the images that were quantified with GliaMorph.

Figure 1 – clear overview figure that clearly defines the question being addressed in the manuscript. Would be even better if followed by Figure 10.
Placement of the transgenic descriptions on 1A' are partially obscured due to white writing and white signal from the glia.
The sentence from lines 1242-1244 does not belong in the figure caption How was the schematic in B generated?
Line 1248 should have C'
The significance of the computational descriptions shown in C'' wasn't made clear to me in the results or discussion section.

Nyquist sampling needs to have specific values with it so that the non-expert reader can better understand the changes in z-step for imaging.

Figure 2 – The subregion tool provides a way to select similar parts of different images and seems useful for many different tissues, not just the retina.
For part A, some of the information in Supplemental Figure 2, especially the images in part E are important for understanding how the bounding boxes are drawn and then aligned among the images. The authors could consider revising Figure 2 to include these or something like them.
For part B, the 24 hpf image shows only a small amount of the Tg[TP1bglob:GFPcaax] transgenic signal. Is this representative of all of the images at this time point? From the way the figure caption and results are written, I think, but am not sure that the overlaid images are from separate fish. Please clarify.
For part C, what exactly is measured as progenitor and MG height? Which transgenic lines were used for this measurement?

Figure S3 – deconvolution. In my naiveté, I have always thought that confocal micrographs, especially those captured with a relatively small pinhole (1.2 Airy units or smaller), did not need deconvolution. In fact, all but one of the deconvolution algorithms used in this study generated images that were less resolved than the original. Application of a single iteration of the Richardson-Lucy deconvolution algorithm appeared to give some minimal improvement in resolution/sharpness of the image, but is this a necessary step? If it is a necessary step, it seems odd to confine all of these data to supplemental figures.

Minor point about part S3H: I was confused about the different number of dots for the different cellular features. For instance, there only two dots for the cell body quantification of the original image but six for the other cellular features? Are the dots simply overlapping? If so, a small amount of jitter for the points could help.

Figure S4. The Airyscan image analysis doesn't really seem to add much especially since the analysis performed are part of the Zeiss package. As the paper is long and seems to struggle with exactly what type of paper it is – Methods, Description of construction of image analysis tool, or Application of tool for biological insights – I would encourage the authors to streamline this section of the results or remove it.

Figure 3 – One of the most important of the figures in the paper. The ZonationTool seems powerful, not just for MGs but for any other cell type in an epithelium, especially a neuroepithelium. Therefore, I would recommend that its details be explained with more clarity. It took me a hot second to realize that part B is actually describing what the ZonationTool does to be able to make the plots shown in part C and along the side of the image in part A.
Figure caption needs information that addresses the following questions:
-Do the numbers along the left side of the heat maps in parts E and H represent absolute position along the apicobasal axis?
-Are the heat map images in E showing MG on the left and RGCs on the right?
-I'm guessing that CB = cell body and EF = endfoot but it would be nice to have the abbreviations listed and defined in the figure caption.
-In part H, do the heat maps representing the means in G (not H)?

© 2023. Published by The Company of Biologists under the terms of the Creative Commons Attribution License (https://creativecommons.org/licenses/by/4.0/).

Figure 4 – shows the morphological features that GliaMorph can extract. Other clearly describing what is depicted/plotted/measured in the figure caption, this figure seems accurate and important for the main premise of the paper. In fact Figures 3 and 4 together show the usefulness of GliaMorph.

Figure 5 – The figure caption is confusing and needs to describe the figure not tell the results. In particular, on line 1318, the authors write: "(D-M) All parameters, except MG height, did not significantly change during the studied time frame", but then in for part I and part M, the authors suggest that the thickness and average branch length were statistically significantly increased. Not only is this information contradictory, it should be in the results section.

Figure S6 – In my view, the results and/or discussion would benefit from a more explicit comparison of Figures 4 and 5. The authors indicate that the features extracted with the membrane marker provide a higher level of resolution/detail than a cytoplasmic marker, but only cite supplemental Figure 6 instead of doing some sort of more global comparison of Figures 4 and 5. Moreover, if is my view that including some or all of Figure S6 in the main figures, perhaps combining it with Figure 7, would be beneficial as it shows the importance of mosaic analysis (which isn't necessarily surprising, but an important caveat when using this Tool).

Figure 6 – again the figure caption and/or methods need more detail about what is actually shown and how the various plots were made. For what it's worth, I found the comparison between Figure 5 and Figure 6 informative.

Figure 7 – shows that single-cell analysis shows more statistically significant morphological changes over time compared to bulk analysis. This is similar/complementary to what has been described for MG that are observed over the course of regeneration experiments and during classic retinal development studies. As with other figures, the caption needs to report what is shown not report results and/or conclusions.

The data presented in Figures 8 and S7 should be removed. I have strong reservations about these F0 crispant studies. It is my view that without additional controls showing which cells in the retina have a loss of cdh2 expression, the data cannot be robustly interpreted. Have other crispant analyses been performed that could be used to estimate the number of cells that are targeted/altered with the injections? Performing experiments to fluorescently mark the cells that are likely to have changes in the cdh2 locus? One possibility would be to engineer a fluorescent reporter that shows Cas9-mediated cutting. Another possibility would be to perform in situ hybridization or immunohistochemistry to determine which cells/regions of the retina have lost cdh2 expression. Minimally controlled CRISPR/Cas9 experiments that do not generate stable lines, like the one performed in this study, can lead to misinterpretation of results due to off-target and mosaic effects. Although the authors do include a note of caution, these data actually detract from the paper rather than add to it.

Figure 9 – data from mouse models of retinal disease are a nice addition to this proof-of-concept paper. Again the figure caption needs to report what is shown not the results/conclusions of the data.

Figure 10 – a super helpful figure that, in my view, needs to be moved toward the front of the manuscript to help guide the reader through the study.

Reviewer 2

*Advance summary and potential significance to field*

In this resourceful paper by Kugler et al., a suite of image analysis tools have been provided to quantitatively assess Müller glial cell morphology. The authors use their pipeline to provide detailed information about the changing morphology of Müller glia across developmental ages in the zebrafish retina, in different species – zebrafish and mouse, and in pathological conditions (in the absence of cadherin2 and in a mouse glaucoma model). I anticipate that this tool will be well received and implemented by the Developmental Biology community, as researchers beyond those

© 2023. Published by The Company of Biologists under the terms of the Creative Commons Attribution License (https://creativecommons.org/licenses/by/4.0/).

studying Müller cells could adapt it for their cell-type of interest. While the authors don't explicitly test their tools to probe neurons or other complex cells in the retina or brain, it seems reasonable to expect that GliaMorph should be applicable here too. The authors clearly make this analysis tool set available to the community, including the code on Github.

*Comments for the author*

The comments below are minor where clarifications and corrections suffice

Figure 1B – Is this really a schematic as the authors write or max. intensity projections of individual cells at distinct ages?

Fig 1- Is C'' meant to be computationally extracted content from C' or a 'schematic' representation of C'.

In lines 197-198 – the authors write that they chose a standard region for analysis as cell morphologies vary with location in the retina – I assume they refer to neurons here or do they mean there are morphological differences of Muller glia across the retina?

Fig 3H – INL on the heat map appears twice.

It is unclear to me why the #Endpoints differs so drastically between Fig 4N, Fig 5L. and Fig 7H for the 72 hpf time-point.

Fig 7A Why do the authors refer to these as clones rather than individual cells?

Fig S1 C' shouldn't the distance (x-axis) be displayed in µm?
Fig S1 D,E wouldn't the benefits of an optimal z-step be better displayed in x-z rather than a max. intensity projection?

---

**First revision**

<u>Author response to reviewers' comments</u>

**Point-by-point response to reviewers:**

Reviewer 1 Advance Summary and Potential Significance to Field:
This study describes GliaMorph, a semi-automated, non-deep learning approach to quantifying morphological parameters of Müller glia in the vertebrate retina called. It is a companion to a detailed methods paper submitted elsewhere (and supplied in the supplemental files here) and accompanied by a series of YouTube video tutorials for implementing GliaMorph.

Standardized image analysis approaches that include steps such as feature extraction and the ability to reliably compare images across samples/labs and conditions is a current and growing field within cell and developmental biology. As more and more image data is standardized and protocols for depositing raw image data are developed (and required) approaches like the one described in this paper will become commonplace. This manuscript includes information about the specific approaches the researchers took to build GliaMorph. The authors give an overview of the steps required to establish and then apply GliaMorph, showing that the approach can detect features including positioning of nuclei and other cellular features along the apicobasal axis, general cell morphology features including branch points, cell body size, and skeletal shape. In general, GliaMorph appears to be a useful approach for reproducible image analysis of M. The authors also apply GliaMorph to other samples to test whether it can detect differences in Müller glia cells across different conditions.
Because this manuscript describes a new tool and validates it, but does not contribute a significant biological conceptual advance, I would suggest that this paper could be considered as a Resource/Technique rather than a Research article as the novelty is in the method and approach rather than the biological findings.

© 2023. Published by The Company of Biologists under the terms of the Creative Commons Attribution License (https://creativecommons.org/licenses/by/4.0/). 

**Our response:** We thank the reviewer for their comments and hope that the revisions address their feedback sufficiently. We have significantly streamlined the manuscript to focus on the implementation of the tool and highlight the biological insights gained from GliaMorph. This includes a substantial reorganisation of multiple sections which have been highlighted in the revised version, rephrasing of Results section and figure titles and revising figure legends to comply with Development standards. We would be happy for the manuscript to be considered as a Resource/Technique article if the editor feels this is the most appropriate category. Again, many many thanks for your time and attention to improve our work.

**Reviewer 1 Comments for the Author:**

I enjoyed reading this manuscript and previewing GliaMorph. Below are a number of suggestions for improving the readability of the manuscript as well as ensuring the accuracy of the study.

1. Overall organization of the paper could be improved with a number of changes including considering how to organize the figures so that they are discussed in the order in which they appear in the manuscript and by moving figure 10 closer to the beginning of the manuscript (possibly after Figure 1). Moving Figure 10 up will provide a roadmap for the reader, using it to set up the flow of the paper which essentially walks through how each part of the workflow was established and then validates the approach by analyzing MG morphology over time and assessing retinal disease mouse tissue sections.

**Our response:** We thank the reviewer for this very helpful suggestion. We have now moved Fig. 10 to become Fig. 1 to provide a better roadmap for the reader. We have then followed this roadmap to describe how each step was established and what biological insights they provide.

2. Some thoughts about statistics and the plots. It's been my understanding that the Kruskal-Wallis test is used when you have a ranked variable and a nominal variable. It's unclear to me which is the ranked variable in the analyses (maybe the developmental stages). The Kruskal-Wallis test is NOT a test of the difference of means or medians, rather the null hypothesis that is tested with the Kruskal-Wallis test is that the *mean ranks* of the groups are the same. The expected mean rank depends on the total number of observations and isn't as representative of the distribution (and means/medians) of the data. The Mann-Whitney-Wilcoxon test is the non-parametric version of a two-tailed t-test and is similar to the Kruskal-Wallis test. It may or may not be the most appropriate statistic as it is unclear whether the data were tested for the shape of their distribution. The majority of the plots show, I assume, mean and standard deviation, but what exactly is plotted needs to be more clearly indicated in the figure captions.

**Our response:** Thank you for bringing this to our attention. We hope we have understood your concerns and have discussed these thoughts on the statistical tests with several experts in the field of statistical analysis. To our understanding, the Mann-Whitney- Wilcoxon is applied when two groups are tested, while Kruskal-Wallis test is applied where there are more than 2 groups. We did examine data distributions and their shape (https://www.scribbr.com/statistics/statistical-tests/), as stated in the Materials and Methods. As such, we feel these tests are the most appropriate for the data represented here. We apologise that it was not clear what was plotted, we have now re-written the figure legends to clearly state means and standard deviations are plotted.

3. The separation of the Results from much of the Methods section, particularly the sections of Image Analysis through Statistical Analysis, does not work so well for this particular manuscript. From my perspective, the Methods for all the steps of GliaMorph are integral to, if not actually part of the Results for this study. This speaks to the larger issue with this manuscript - as it seems that this manuscript is a validation/application of GliaMorph, providing a way to more systematically and quantifiably describe the features previously described for Müller Glia. It is my view that with extensive rewriting/revision that streamlines that study and focuses on the insights gained from the implementation of the tool (such as those described in Figures 3-6) could improve the impact of the paper.

**Our response:** We thank the reviewer for this comment and hope they agree our re- structed and streamlined version focuses on the insights gained by employing GliaMorph. While we made a concerted effort to highlight the insights gained, we did feel in certain instances the steps and rationale for developing certain tools needed to be explained in the results section ("Establishing

© 2023. Published by The Company of Biologists under the terms of the Creative Commons Attribution License (https://creativecommons.org/licenses/by/4.0/).

image comparability and reproducibility to reliably quantify MG cells in the retina" Lines 199-234). However, these sections are greatly reduced in comparison to the original version. This is a challenging line to walk as this manuscript is an interdisciplinary study between biomedical image analysis applied to biology and want to highlight that our work includes benchmarking, internal controls and proof-of-principle data. As such, we hope the editor and reviewer appreciate the significantly revised manuscript reframed around the insights gained by each GliaMorph Tool.

4. The analysis of cdh2 crispants should be removed. This part of the manuscript detracts from the overall message of the paper and isn't as robust as the data from mice retinae. I also have some technical concerns with this experiment and those are detailed below.

**Our response:** We thank the reviewer for this comment and agree. As such, we have removed the cadherin crispant sections in the manuscript and corresponding Figure 8 and Figure S7. We will conduct further control experiments and use this data in a future publication.

5. Images are oriented with apical up and basal down which is fine, but in the ophthalmology/clinical fields basal is up and apical is down. Moreover, when embryologists orient images, the convention is typically dorsal as up and ventral down. Maybe a note about the orientation of the images somewhere prominent and/or early in the results would be helpful for all readers.

**Our response:** We appreciate the reviewers comment and acknowledge there is no standardised orientation for retinal sections. There appears to be a clear discrepancy between developmental and clinical studies. We clarified the difference in representation in models, including clearly stating the orientation in figure legends ("In all images apical is the top and basal is the bottom of the image.") and the materials and methods (Lines 679- 681).

6. All figure captions need to describe the figures not report results. The running title for each figure caption can summarize the results of the figure, but what is in the figure should be described. For example, for the plots with data points, instead of writing that there was or wasn't a statistically significant difference, describe the axes and the type of plot and what the data points represent.
**Our response:** Thank you for pointing this out. We have now adapted all figure captions to describe the figure and highlight the biological significance of the findings. We have also shortened the legends by reducing repetitive text and maintain their adherence to journal standards.

7. While Müller glia definitely have some features of astrocytes, they also bear resemblance to radial glia, especially in organisms that can regenerate and/or grow continuously.
**Our response:** We completely agree with the reviewer. In fact, we believe radial glia may be the most useful cell type to study with GliaMorph besides MG. We have now clarified this in Line 116-117 in the introduction and Line 526 in the discussion.

8. There are many additional useful resources/companions to this manuscript. The YouTube videos for implementation and the written protocol should be prominently cited to improve the likelihood that this tool will be widely used.
**Our response:** Many thanks for pointing this out. We included respective pointers in the "Workflow integration" (Lines 389-393) and "Code and data availability" section (Line 893- 898).

Specific comments:
Methods:
Construct generation: need more details/references for the plasmids and Multisite Gateway technology. Did the authors use purified components from their own lab or did they purchase a kit? Does the pME-mCherry-CAAX plasmid have a reference or a way that others could obtain it?
**Our response:** Thank you for pointing this out. These techniques are considered standard in the zebrafish community but we appreciate this may not be widely known by researchers in other fields. We have now included additional details and references to clearly describe how we generated the plasmids for this study (Lines 661-671).

© 2023. Published by The Company of Biologists under the terms of the Creative Commons Attribution License (https://creativecommons.org/licenses/by/4.0/).

Cdh2 CRISPR/Cas9 injections: How was the efficacy of CRISPR/Cas9 evaluated? What controls were used to confirm specificity?
**Our response:** We completely agree with the reviewer that this data is too preliminary for inclusion in the current manuscript. We have now completely removed the cadherin crispant sections and figures as suggested by the reviewer.

Fixation: Dehydration was performed by consecutive 5-minute washes with 25%, 50%, 75% Methanol (MeOH) in PBS. Instead of “Dehydration was performed by consecutive 5 minute washes with 25%, 50%, 75% in PBS....”
**Our response:** Thank you for pointing this out. We have now corrected this (Lines 598- 599).

Immunohistochemistry: DAPI is 4′,6-diamidino-2-2 phenylindole, not 4′,6- diamidino-2- 532 phenylindole. DAPI staining typically follows immunohistochemistry. What was the stock concentration of DAPI? Was it diluted from a stock that was made in DMSO or PBS?
**Our response:** We have now addressed this comment in an updated “Zebrafish Immunohistochemistry” section (Lines 614-617).

What was the temperature for acetone incubation?
**Our response:** We have now included this information, that this was conducted at -20°C (line 608).

Image Analysis and on:
Line 750 – CNR abbreviation is used before it is defined on line 762
**Our response:** Thank you for pointing this out. We’ve corrected this.

Lines 751-754 seem superfluous since the main point of the paper is the new image analysis method.
**Our response:** We thank the reviewer for the comment but while these details may seem superfluous, the descriptions on sampling, blinding, and bias are according to ARRIVE guidelines. As such, we did not remove or change these. We hope the reviewer will agree that these are important to comply with ARRIVE and journal standards.

Results with a focus on Figures and Tables:
Table 1 needs some footnotes to be more readily understood. For example, I’m assuming that A-B stands for apical to basal, but that needs to be explicit and some explanation about how exactly radial applies to microglia and astrocytes. What is the significance of the two colors?
**Our response:** Many thanks for pointing out the lack of clarity. We have amended this accordingly.

Table 2 seems out of place in the methods; it seems more like part of the Results since it is describing the features of the images that were quantified with GliaMorph.
**Our response:** We appreciate the reviewer’s comment, but we have also described the quantified features throughout the text (e.g. section “3D feature extraction reveals MG sub- cellular elaboration during development” Lines 261-284). We included Tables 2 and 3 in the Material and Methods (now corrected numbering), as this is customary in image analysis and aids the description of methods used and referred to them in the main body of the text (Line 240 and Line 276).

Figure 1 – clear overview figure that clearly defines the question being addressed in the manuscript. Would be even better if followed by Figure 10.
**Our response:** We thank the reviewer for this helpful suggestion and have now addressed this by moving Fig 10 to Fig 1. We now follow this workflow in the description of the development and implementation of the tool throughout the manuscript.

Placement of the transgenic descriptions on 1A’ are partially obscured due to white writing and white signal from the glia.
**Our response:** Thanks for pointing this out. We addressed this by moving the labels of Fig 1A’ next to the images and making them black.

The sentence from lines 1242-1244 does not belong in the figure caption
**Our response:** We’ve rewritten this section of the legend and hope the reviewer agrees this is clearer in the revised version of the manuscript (Now figure 2 – Lines 1095-1107).

© 2023. Published by The Company of Biologists under the terms of the Creative Commons Attribution License (https://creativecommons.org/licenses/by/4.0/).

How was the schematic in B generated?
**Our response:** The schematic was hand drawn, based on observed biological data - we have clarified this in the revised version (Line 1105).

Line 1248 should have C'
**Our response:** Thank you and corrected.

The significance of the computational descriptions shown in C'' wasn't made clear to me in the results or discussion section.
**Our response:** Thank you for pointing this out and we have now elaborated on the importance in the respective results section (Lines 183-188).

Nyquist sampling needs to have specific values with it so that the non-expert reader can better understand the changes in z-step for imaging.
**Our response:** We added the respective information in the Material and Methods section "Sampling Frequency" (Lines 637-649).

Figure 2 – The subregion tool provides a way to select similar parts of different images and seems useful for many different tissues, not just the retina.
For part A, some of the information in Supplemental Figure 2, especially the images in part E are important for understanding how the bounding boxes are drawn and then aligned among the images. The authors could consider revising Figure 2 to include these or something like them.
**Our response:** We agree with the reviewer that this tool can be applied to images of many different tissues. We have now included a specific point in the discussion to draw attention to this fact (Lines 512-516). However, while the bounding box establishment appears simple at first, computationally this is rather complex. We initially circulated the manuscripts with the bounding box information included and received feedback that this was "too computational" and distracting from the biology. We thus created the now Fig. 3A as an overview and refer the interested reader to the supplementary. We hope the reviewer agrees that this is in line with your suggestion to make this manuscript more focused on its biological impact and meaning. As such, we did not include the bounding boxes in the main figure 3 (previously 2).

For part B, the 24 hpf image shows only a small amount of the Tg[TP1bglob:GFPcaax] transgenic transgenic signal. Is this representative of all of the images at this time point? From the way the figure caption and results are written, I think, but am not sure that the overlaid images are from separate fish. Please clarify.
**Our response:** Yes, you are correct. The 24hpf is very dim at this age and very little cell information can be seen at the posterior part of the retina where we acquire data. We have now clarified that these are images from different samples and representative images in the figure legend (Lines 1117-1118).

For part C, what exactly is measured as progenitor and MG height? Which transgenic lines were used for this measurement?
**Our response:** We have now clarified that the distance we measure is GCL-to-OPL and measured in Tg(TP1bglob:GFPcaax) (Lines 1118-1119) in the results section (Line 248- 249).

Figure S3 – deconvolution. In my naiveté, I have always thought that confocal micrographs, especially those captured with a relatively small pinhole (1.2 Airy units or smaller), did not need deconvolution. In fact, all but one of the deconvolution algorithms used in this study generated images that were less resolved than the original. Application of a single iteration of the Richardson-Lucy deconvolution algorithm appeared to give some minimal improvement in resolution/sharpness of the image, but is this a necessary step? If it is a necessary step, it seems odd to confine all of these data to supplemental figures.
**Our response:** Unfortunately, PSF and deconvolution are highly complex topics, and depend on a myriad of factors: We've shared relevant information from the "confocal laser scanning tutorial" below: https://www.med.unc.edu/microscopy/wp-content/uploads/sites/742/2018/06/clsm-tutorial-v2.pdf . "The size of the Airy unit at the pinhole depends on the objective lens NA, the wavelength of the fluorescent light, and any magnification up to the pinhole" "1) For pinhole diameters less that about 1/2 Airy unit, the strength of sectioning (Z resolution) remains constant. 2) For pinhole diameters greater than one Airy unit, the Z resolution slowly becomes worse. 3) A

© 2023. Published by The Company of Biologists under the terms of the Creative Commons Attribution License (https://creativecommons.org/licenses/by/4.0/).

pinhole diameter approximately 1/5 Airy unit will produce maximum lateral resolution at the expense of a loss of 95% of the signal. 4) Lateral resolution is more sensitive to pinhole size than axial resolution and rapidly becomes worse as the pinhole is made larger than one Airy unit."

However, or maybe because of this, we think that keeping this section is critical to emphasize the importance of data exploration and pre-processing. As this topic is highly complex, we cannot possibly do it full justice and this is why expanded on the section to explain that our presented data exploration for deconvolution is a steppingstone (especially if wanting to refocus on the biological insights). Please see the relevant section in the manuscript for further details. We renamed the section to further clarify this ***"Establishing image comparability and reproducibility to reliably quantify MG cells in the retina"*** (Lines 226- 234) and discussed in Lines 449-457.

Minor point about part S3H: I was confused about the different number of dots for the different cellular features. For instance, there only two dots for the cell body quantification of the original image but six for the other cellular features? Are the dots simply overlapping? If so, a small amount of jitter for the points could help.
**Our response:** Yes, these dots are simply overlapping. As we want to show differences due to deconvolution using lines. We apologise for the confusion

Figure S4. The Airyscan image analysis doesn't really seem to add much, especially since the analysis performed are part of the Zeiss package. As the paper is long and seems to struggle with exactly what type of paper it is – Methods, Description of construction of image analysis tool, or Application of tool for biological insights – I would encourage the authors to streamline this section of the results or remove it.
**Our response:** We agree with the reviewer and have streamlined these section in the results. Please also see above.

Figure 3 – One of the most important of the figures in the paper. The ZonationTool seems powerful, not just for MGs but for any other cell type in an epithelium, especially a neuroepithelium. Therefore, I would recommend that its details be explained with more clarity. It took me a hot second to realize that part B is actually describing what the ZonationTool does to be able to make the plots shown in part C and along the side of the image in part A.
Figure caption needs information that addresses the following questions:
-Do the numbers along the left side of the heat maps in parts E and H represent absolute position along the apicobasal axis?
-Are the heat map images in E showing MG on the left and RGCs on the right?
-I'm guessing that CB = cell body and EF = endfoot but it would be nice to have the abbreviations listed and defined in the figure caption.
-In part H, do the heat maps representing the means in G (not H)?

**Our response:** We agree with the reviewer of the importance of this figure and thank them for the suggestions in clarifying. We have addressed all comments accordingly in the figure (now Fig 4), Results (Lines 237-259; 280-284; 371-373) and Discussion (Line 412- 415; 431-437; 476-477). We've also clarified the caption for Figure 4 to address the reviewers concerns (Lines 1143-1144).

Figure 4 – shows the morphological features that GliaMorph can extract. Other clearly describing what is depicted/plotted/measured in the figure caption, this figure seems accurate and important for the main premise of the paper. In fact, Figures 3 and 4 together show the usefulness of GliaMorph.
**Our response:** Thank you for the recognition of the importance of these figures (now Fig 4 and 5). We agree and have now modified the legend to shorten it and increase clarity. We have also discussed the insights gained by these tools throughout the discussion (Lines 397-419).

Figure 5 – The figure caption is confusing and needs to describe the figure not tell the results. In particular, on line 1318, the authors write: "(D-M) All parameters, except MG height, did not significantly change during the studied time frame", but then in for part I and part M, the authors suggest that the thickness and average branch length were statistically significantly increased. Not only is this information contradictory, it should be in the results section.

© 2023. Published by The Company of Biologists under the terms of the Creative Commons Attribution License (https://creativecommons.org/licenses/by/4.0/).

**Our response:** Thank you for pointing this out. We have now removed the contradictory statement and corrected the text to reflect the data showing increased and decreased parameters (Lines 336-339 and legend; Lines 1168-1180).

Figure S6 – In my view, the results and/or discussion would benefit from a more explicit comparison of Figures 4 and 5. The authors indicate that the features extracted with the membrane marker provide a higher level of resolution/detail than a cytoplasmic marker, but only cite supplemental Figure 6 instead of doing some sort of more global comparison of Figures 4 and 5. Moreover, if is my view that including some or all of Figure S6 in the main figures, perhaps combining it with Figure 7, would be beneficial as it shows the importance of mosaic analysis (which isn't necessarily surprising, but an important caveat when using this Tool).
**Our response:** In the revised version of the manuscript, we compared the now figure 5 and figure 6 as stated above. Additionally, we expanded the discussion (Lines 397-419).

Figure 6 – again the figure caption and/or methods need more detail about what is actually shown and how the various plots were made. For what it's worth, I found the comparison between Figure 5 and Figure 6 informative.
**Our response:** Thank you for pointing this out. We rewrote the image description in the legend (now Fig 7) to increase details of what we show in the legend (Lines 1182-1192) and Discussion (Lines 405-409).

Figure 7 – shows that single-cell analysis shows more statistically significant morphological changes over time compared to bulk analysis. This is similar/complementary to what has been described for MG that are observed over the course of regeneration experiments and during classic retinal development studies. As with other figures, the caption needs to report what is shown not report results and/or conclusions.
**Our response:** We agree with the reviewer and thank them for recognising the previously published morphological changes observed in development/regeneration are reflected in our analysis. However, to the best of our knowledge, there has not been a quantification of just how much more detail is gained from single cell vs bulk analysis. In the case of MG it is significant. We have now updated the legend to report the results more clearly and concisely (Lines 1195-1109) and Discussed on Lines 413-419.

The data presented in Figures 8 and S7 should be removed. I have strong reservations about these F0 crispant studies. It is my view that without additional controls showing which cells in the retina have a loss of cdh2 expression, the data cannot be robustly interpreted. Have other crispant analyses been performed that could be used to estimate the number of cells that are targeted/altered with the injections? Performing experiments to fluorescently mark the cells that are likely to have changes in the cdh2 locus? One possibility would be to engineer a fluorescent reporter that shows Cas9- mediated cutting. Another possibility would be to perform in situ hybridization or immunohistochemistry to determine which cells/regions of the retina have lost cdh2 expression. Minimally controlled CRISPR/Cas9 experiments that do not generate stable lines, like the one performed in this study, can lead to misinterpretation of results due to off-target and mosaic effects. Although the authors do include a note of caution, these data actually detract from the paper rather than add to it.
**Our response:** We completely agree with the reviewer. As such we have completely removed the cadherin crispant sections in the manuscript and removed Figure 8 and Figure S7.

Figure 9 – data from mouse models of retinal disease are a nice addition to this proof-of-concept paper. Again the figure caption needs to report what is shown not the results/conclusions of the data.
**Our response:** Thank you for the comment. We have altered the figure caption accordingly and added additional axis information in the figure to support the data interpretation (Lines 1214-1222).

Figure 10 – a super helpful figure that, in my view, needs to be moved toward the front of the manuscript to help guide the reader through the study.
**Our response:** We agree with the reviewer and have now moved this to Figure 1.

© 2023. Published by The Company of Biologists under the terms of the Creative Commons Attribution License (https://creativecommons.org/licenses/by/4.0/). 

Reviewer 2 Advance Summary and Potential Significance to Field:
In this resourceful paper by Kugler et al., a suite of image analysis tools have been provided to quantitatively assess Müller glial cell morphology. The authors use their pipeline to provide detailed information about the changing morphology of Müller glia across developmental ages in the zebrafish retina, in different species – zebrafish and mouse, and in pathological conditions (in the absence of cadherin2 and in a mouse glaucoma model). I anticipate that this tool will be well received and implemented by the Developmental Biology community, as researchers beyond those studying Müller cells could adapt it for their cell-type of interest. While the authors don't explicitly test their tools to probe neurons or other complex cells in the retina or brain, it seems reasonable to expect that GliaMorph should be applicable here too. The authors clearly make this analysis tool set available to the community, including the code on Github.

Reviewer 2 Comments for the Author:
The comments below are minor where clarifications and corrections suffice
**Our response:** We thank the reviewer for their time and comments to improve our manuscript.

Figure 1B – Is this really a schematic as the authors write or max. intensity projections of individual cells at distinct ages?
**Our response:** The schematic was hand drawn, based on observed biological data - we have now clarified this in the revised version (Line 1099).

Fig 1- Is C'' meant to be computationally extracted content from C' or a 'schematic' representation of C'.
**Our response:** Many thanks for pointing our that this wasn't clear. We rewrote this part of the figure legend to clarify this as follows: "Manually drawn schematic of how MG subregions features could be described for computational analysis in terms of shape." (Line 1105-1106).

In lines 197-198 – the authors write that they chose a standard region for analysis as cell morphologies vary with location in the retina – I assume they refer to neurons here or do they mean there are morphological differences of Muller glia across the retina?
**Our response:** We expanded on this to clarify our intention as follows: "To standardize the ROI for image acquisition, we focus on the ventro-temporal retina, as regional differences in anatomy (e.g., high acuity area vs periphery) and cell morphologies (e.g., photoreceptor neurons) exist across the retina in zebrafish (**Fig 2A**) [24], [25]." (Lines 175-178).

Fig 3H – INL on the heat map appears twice.
**Our response:** Thank you for pointing this out. In the revised version this is Fig 4H, and we corrected this mistake accordingly.

It is unclear to me why the #Endpoints differs so drastically between Fig 4N, Fig 5L. and Fig 7H for the 72 hpf time-point.
**Our response:** Thank you very much for pointing out what we clearly overlooked. Looking back through the data, we ran the quantificationTool on the data for now Figure 5 (previous Fig. 4) using a previous code version. Re-running the analysis with the most previous version showed that the data we had in the previous manuscript version were incorrect due to changes in the level of skeleton pruning. We rectified all skeleton-related data in Figure 5 accordingly.

Fig 7A Why do the authors refer to these as clones rather than individual cells?
**Our response:** This is a great point by the reviewer. These were referred to clones because these single MG cells are often born from an individual retinal progenitor cell, which has been shown in previous experiments from other laboratories. However, as sometimes clones can result in more than one cell being labelled, we have now completely removed the references to "clones" and clarified it to "single-cell" analysis. We have also added more information in the Material and Methods section "Constructs Generation" (Lines 570-581) to how we generated the single-cell MG labelled cells. We hope this clarifies the point.

Fig S1 C' shouldn't the distance (x-axis) be displayed in µm?
**Our response:** Yes, we corrected this.

© 2023. Published by The Company of Biologists under the terms of the Creative Commons Attribution License (https://creativecommons.org/licenses/by/4.0/).

Fig S1 D,E wouldn't the benefits of an optimal z-step be better displayed in x-z rather than a max. intensity projection?
**Our response:** We agree and have now added a panel to show the z-axis in addition to the MIP, which shows the difference in z-steps better.

---

Second decision letter

MS ID#: DEVELOP/2022/201008

MS TITLE: GliaMorph: A modular image analysis toolkit to quantify Müller glial cell morphology

AUTHORS: Elisabeth C Kugler, Isabel Bravo, Xhuljana Durmishi, Stefania Marcotti, Sara Beqiri, Alicia Carrington, Brian C Stramer, Pierre Mattar, and Ryan B MacDonald
ARTICLE TYPE: Research Article

I am happy to tell you that your manuscript has been accepted for publication in Development, pending our standard ethics checks.

Reviewer 1

*Advance summary and potential significance to field*

This study describes GliaMorph, a semi-automated, non-deep learning approach to quantifying morphological parameters of Müller glia in the vertebrate retina and is accompanied by a series of YouTube video tutorials for implementing GliaMorph for cells with the same or similar morphologies.

Standardized image analysis approaches that include steps such as feature extraction and the ability to reliably compare images across samples/labs and conditions is a current and growing field within cell and developmental biology.
As more and more image data is standardized and protocols for depositing raw image data are developed (and required) approaches like the one described in this paper will become commonplace. In general, GliaMorph appears to be a useful approach for reproducible image analysis. As this manuscript is describing a new tool and validating it, but not, in my view, contributing a significant biological conceptual advance, I would suggest that this paper would fit in the Resource/Technique section of Development.

*Comments for the author*

The authors have addressed all of my concerns. Thank you!

Minor comments:
-umlaut u converted weirdly in the pdf to a box
-sentence on line 461 should begin "Again, it is important..."

Reviewer 2

*Advance summary and potential significance to field*

The manuscript by Kugler et al., provides an image analysis pipeline to quantitatively assess Müller glial cell morphology. Their tool set should be broadly applicable to the assessment of other complex cells.
In the revised version of the manuscript, the authors responded to all my queries satisfactorily and further made many changes to the original manuscript, which in my opinion, greatly improves it.

© 2023. Published by The Company of Biologists under the terms of the Creative Commons Attribution License (https://creativecommons.org/licenses/by/4.0/). 

*Comments for the author*

The authors have done a very thorough job of responding to and accordingly revising, adding or clarifying my queries and that of the other reviewer.
The rearrangement of some material, including figures, make the revised manuscript much improved.

© 2023. Published by The Company of Biologists under the terms of the Creative Commons Attribution License (https://creativecommons.org/licenses/by/4.0/).
